# Nocturnal oxygen therapy in obstructive sleep apnoea: a systematic review and meta-analysis

**DOI:** 10.1183/16000617.0173-2023

**Published:** 2024-03-20

**Authors:** Su Latt Phyu, Selin Ercan, Eli Harriss, Christopher Turnbull

**Affiliations:** 1 Oxford Centre for Respiratory Medicine, Oxford University Hospitals NHS Foundation Trust, Oxford, UK; 2 Department of Internal Medicine and Clinical Nutrition, Krefting Research Centre, University of Gothenburg, Goteborg, Sweden; 3 Bodleian Health Care Libraries, University of Oxford, Oxford, UK; 4 University of Oxford, NIHR Oxford Biomedical Research Centre, Oxford, UK; 5 Both authors contributed equally to this work

## Abstract

Obstructive sleep apnoea is characterised by recurrent reduction of airflow during sleep leading to intermittent hypoxia. Continuous positive airway pressure is the first-line treatment but is limited by poor adherence. Nocturnal oxygen therapy may be an alternative treatment for obstructive sleep apnoea but its effects remain unclear. This meta-analysis evaluates the effects of nocturnal oxygen therapy on both obstructive sleep apnoea severity and blood pressure.

A literature search was performed based on the Preferred Reporting Items for Systematic Review and Meta-analysis guidelines. Peer-reviewed, randomised studies that compared the effect of nocturnal oxygen therapy to sham in obstructive sleep apnoea patients were included. The main outcomes were the apnoea–hypopnoea index and systolic and diastolic blood pressure.

The search strategy yielded 1295 citations. Nine studies with 502 participants were included. When nocturnal oxygen therapy was compared to sham/air, it significantly reduced the apnoea–hypopnoea index (mean difference (MD) −15.17 events·h^−1^, 95% CI −19.95– −10.38 events·h^−1^, p<0.00001). Nocturnal oxygen therapy had no significant effect on blood pressure at follow-up without adjustment for baseline values, but did, where available, significantly attenuate the change in blood pressure from baseline to follow-up for both systolic blood pressure (MD −2.79 mmHg, 95% CI −5.45– −0.14 mmHg, p=0.040) and diastolic blood pressure (MD −2.20 mmHg, 95% CI −3.83– −0.57 mmHg, p=0.008).

Nocturnal oxygen therapy reduced the apnoea–hypopnoea index severity and the change in (but not absolute) systolic and diastolic blood pressure, compared to sham. This suggests that nocturnal oxygen therapy may be a treatment option for obstructive sleep apnoea. Further studies with longer-term follow-up and standardised measurements are needed.

## Tweetable abstract

**In this systematic review and meta-analysis, nocturnal oxygen therapy reduced AHI in the short term in people with OSA but did not reduce blood pressure at follow-up. Studies had small sample sizes and short follow-ups so further research is needed.** https://bit.ly/48hFjGt

## Introduction

Obstructive sleep apnoea (OSA) is a highly prevalent condition affecting approximately one billion people worldwide [1]. It is characterised by recurrent narrowing and/or obstruction of upper airways leading to intermittent hypoxia and sleep disturbance. Untreated OSA is associated with higher cardiovascular risks including hypertension, ischaemic heart diseases and atrial fibrillation [2]. Continuous positive airway pressure (CPAP) treatment is the first-line treatment choice for individuals with OSA syndrome and is highly effective in reversing airway obstruction, thereby improving sleep quality and daytime somnolence [3]. However, its effectiveness is limited by poor tolerance and adherence [4, 5], with randomised controlled trials (RCTs) showing no effect of CPAP on preventing secondary cardiovascular events, albeit with limitations of CPAP usage and excluding patients with the most severe OSA [6].

Nocturnal oxygen therapy (NOT) may be an alternative therapy but the reported effects of NOT are varied. It has been consistently shown to improve oxygenation during sleep [7] but the effects on apnoea–hypopnoea index (AHI) and blood pressure are controversial [8]. We conducted a systematic review and meta-analysis to evaluate the effects of NOT on OSA severity and both systolic blood pressure (SBP) and diastolic blood pressure (DBP) in patients with OSA.

## Methods

The systematic review was performed for all studies until 14 April 2023 and is registered with the international prospective register of systematic reviews (PROSPERO) database (CRD42022316259).

### Eligibility criteria

Peer-reviewed, randomised controlled or crossover studies which investigate NOT compared to sham/control in OSA patients were included.

The study population was adults (≥18 years) with OSA. Studies of patients with predominant central sleep apnoea, obesity hypoventilation syndrome, COPD, OSA–COPD overlap syndrome or other significant respiratory diseases or those with complex interventions such as hypnotic medications were excluded.

### Intervention group

The intervention group comprised OSA patients who received NOT during sleep. Oxygen flow rate and duration of intervention were recorded.

### Control group

Intervention groups were compared with patients who received sham/control with or without healthy lifestyle and sleep education as supportive healthcare.

### Outcome measures

The primary outcome was the effects of NOT on AHI. Secondary outcomes included SBP and DBP (change and follow-up), heart rate (change and follow-up), oxygen desaturation index (ODI), apnoea duration and arousal index. Where available, morning awake blood pressure was selected for blood pressure outcomes.

### Search strategy: identification of studies

The literature search based on Preferred Reporting Items for Systematic Review and Meta-analysis (PRISMA) guidelines was conducted in PubMed, Scopus, Web of Science (Core Collection), Ovid Embase, Cochrane CENTRAL Register of Controlled Trials (first quarter 2022) and the Cochrane Database of Systematic Reviews (all randomised studies until 2022) (E. Harriss). All prospective randomised studies published in English in a peer-reviewed journal until the beginning of 2022 were included. The literature search was updated in April 2023 and identified 101 new references for screening. Reference lists of eligible publications were also screened for retrieval of missing reports. The full search strategy can be found in the supplementary material. De-duplication was performed on the search results.

Screening of the studies was done by two independent reviewers (S. Ercan and S.L. Phyu) using Rayyan software [9] and disagreements were resolved by consensus. If consensus could not be reached, the decision of a third reviewer (C. Turnbull) was sought.

### Data extraction and risk of bias assessment

Data were extracted by two of the authors (S. Ercan and S.L. Phyu). Various data such as authors’ names, publication year, study type, duration of the intervention, oxygen flow rate, number of participants, their mean age, AHI without desaturation, AHI with desaturation, ODI, apnoea duration, arousal index, baseline and follow-up values of SBP and DBP and heart rate were extracted. Mean changes in SBP and DBP were also extracted if provided. If these were not provided, mean changes were calculated by subtracting the baseline values from follow-up data. Standard deviation (sd) of the mean blood pressure changes was calculated using the pooled sd formula:

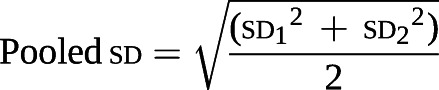


One study recorded mean blood pressure changes instead of giving baseline and follow-up values, which were obtained after contact with the corresponding author. If both per-protocol and intention-to-treat data were available, then the per-protocol analysis data were used. Where values were provided as a median and interquartile range, median values were converted to mean values [10].

Two reviewers (S. Ercan and S.L. Phyu) independently assessed the risk of bias for all the included studies according to the Cochrane Risk of Bias tool (table 1) [20]. Disagreements were resolved through discussion until a consensus was reached.

### Statistical analysis

Review Manager 5.4 (RevMan, Cochrane Review Manager, Cochrane Collaboration, Oxford, UK) was used for the meta-analysis and the random-effects (DarSemonian and Laird method) model was chosen to pool the results. Heterogeneity was assessed based on Chi^2^ tests and I^2^ statistics to evaluate if a sensitivity analysis was needed. Subgroup analyses for oxygen flow rate (≤3 L·min^−1^ *versus* >3 L·min^−1^), study type (randomised crossover *versus* RCT) and intervention duration (1-night trial *versus* longer) were performed to assess their possible effects on the outcomes.

## Results

**FIGURE 1:**
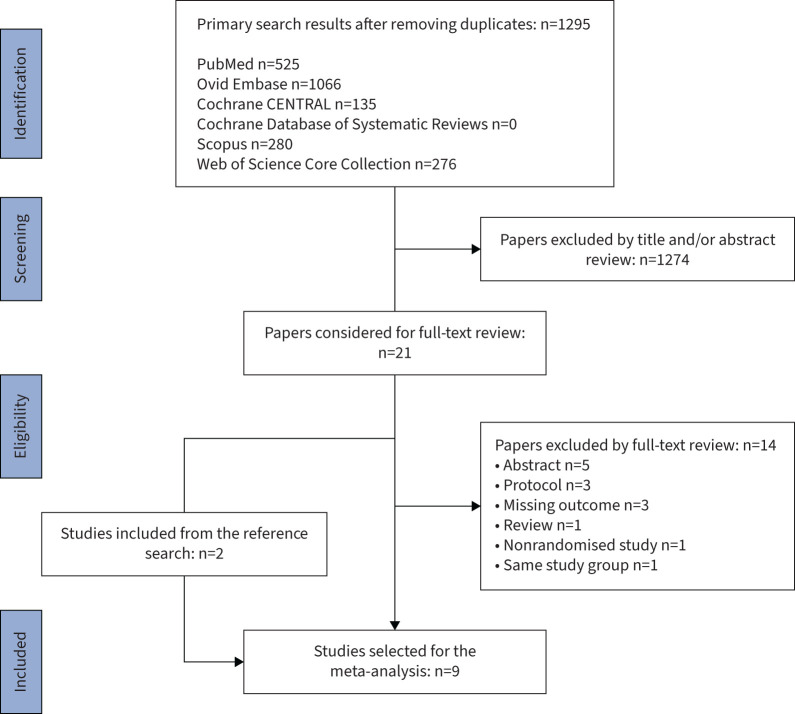
Preferred Reporting Items for Systematic Reviews and Meta-analyses (PRISMA) flow chart of the literature search.

1295 papers were identified with the primary literature search (figure 1). After abstract and full-text review, nine studies with a total of 502 participants were included in the analysis [11–19]. No study was excluded based on the risk of bias assessment (table 1). Four of the included studies were RCTs and five were randomised crossover studies (table 2).

**TABLE 1:** Cochrane Risk of Bias assessment results of the included studies

**Study**	**Randomisation process**	**Bias arising from period and carryover effect**	**Deviations from intended interventions**	**Missing outcome data**	**Measurement of the outcome**	**Selection of reported results**	**Overall bias**
**Phillips *et al.* 1990 [11]**	Some concern	High	Low	Low	Low	Low	High
**Mills *et al.* 2006 [12]**	Some concern	Not applicable	Low	Low	Low	Low	Some concern
**Gottlieb *et al.* 2014 [13]**	Some concern	Not applicable	Low	Low	Low	Low	Some concern
**Liao *et al.* 2017 [14]**	Low	Not applicable	Low	Low	Low	Low	Low
**Sands *et al.* 2018 [15]**	Some concern	Low	Low	Low	Low	Low	Some concern
**Turnbull *et al.* 2019 [16]**	Low	Low	Low	Low	Low	Low	Low
**Beaudin *et al.* 2019 [17]**	Low	Not applicable	Low	Low	Low	Low	Low
**Tan *et al.* 2021 [18]**	Low	Low	Low	Low	Low	Low	Low
**Joosten *et al.* 2021 [19]**	Low	Low	Low	Low	Low	Low	Low

**TABLE 2:** Characteristics of the included studies

**Study**	**Country**	**Funding**	**Study design**	**Intervention**	**Duration of intervention**	**BP measurement procedure**	**Study population included in review, n**	**Baseline data (intervention *versus* control for RCTs)**		
								**Gender, n (male %)**	**Age, years**	**OSA** **, events·h^−1^**
**Phillips *et al.* 1990 [11]**	USA	Sanders Brown Center on Aging, University of Kentucky; NIH General Clinical Research Center	Randomised crossover	Sham *versus* 4 L·min^−1^ NOT *versus* CPAP	4 weeks	Office: mean of 5 nocturnal measurements	8	8 (100)	57±13.6	AHI: 20.5±13.6
**Mills *et al.* 2006 [12]**	USA	NHLBI; UCSD General Clinical Research Center	RCT	Placebo CPAP *versus* CPAP *versus* 3 L·min^−1^ NOT	2 weeks	Office: mean of 3 measurements	NOT: 17 Placebo CPAP: 16	13 (76.5) *versus* 13 (81.3)	43.9±10.3 *versus* 49±10.2	AHI: 61.8±38.8 *versus* 61.2±32.8
**Gottlieb *et al.* 2014 [13]**	USA	NHLBI; National Center for Research Resources	RCT	HLSE *versus* CPAP *versus* 2 L·min^−1^ NOT	12 weeks	Ambulatory	NOT: 94 HLSE: 97	65 (69) *versus* 76 (78)	62.9±7.3 *versus* 63.1±7.7	AHI: 24.0±8.1 *versus* 25.5±8.8
**Liao *et al.* 2017 [14]**	Canada	University Health Network Foundation; Department of Anesthesia, University Health Network-Mount Sinai Hospital, University of Toronto	RCT	Room air *versus* 3 L·min^−1^ NOT	3 nights	NA	NOT: 59 Room air: 44^#^	36 (58) *versus* 43 (70)^¶^	62±10 *versus* 62±12^¶^	AHI: 17.9 (8.8–32.8) *versus* 13.8 (9.1–28.1)^#^
**Sands *et al.* 2018 [15]**	USA	AHA; National Health and Medical Research Council of Australia; R.G. Menzies Foundation; ATS Foundation; Heart Foundation of Australia; Harvard Catalyst	Randomised crossover	Sham oxygen 1-week washout 4 L·min^−1^ NOT	1 night	Office: morning and evening	36	26 (72.2)	55±2	AHI: 57.9±22.1
**Turnbull *et al.* 2019 [16]**	UK	Oxford Radcliffe Hospital Charitable Funds; ResMed UK; NIHR Oxford Biomedical Research Centre	Randomised crossover	Sham oxygen 2 weeks washout with CPAP 5 L·min^−1^ NOT	2 weeks	Both office and home	25	21 (84)	62.7±6.9	ODI (at diagnosis): 48.0 (25.3–68.2)
**Beaudin *et al.* 2019 [17]**	Canada	Alberta Innovates - Health Solutions; Canadian Institutes of Health Research; University of Calgary; Heart and Stroke Foundation of Canada; Natural Sciences and Engineering Research Council of Canada	RCT	Room air *versus* 5 L·min^−1^ NOT	2 weeks	Office: time not specified	NOT: 26 Room air: 26	24 (92.3) *versus* 22 (84.6)	48±8 *versus* 51±8	RDI: 36.5±16.0 *versus* 34.1±11.2
**Tan *et al.* 2021 [18]**	China	National Natural Science Foundation of China	Randomised crossover	Sham oxygen 2 weeks washout 2 L·min^−1^ NOT	1 night	Office: morning	34	34 (100)	47 (44.0–53.0)	ODI: 39.0 (34.2–52.0)
**Joosten *et al.* 2021 [19]**	Australia	AHA; ATS Foundation; NHLBI Grant	Randomised crossover	Sham oxygen 1-week washout 4 L·min^−1^ NOT	1 night	Office: morning	20	17 (85)	51.6±13.6	AHI: 34.0±22.0^+^

Data are presented as n (%), median (25th–75th percentile) or mean±sd. BP: blood pressure; RCT: randomised controlled trial; OSA: obstructive sleep apnoea; NIH: National Institutes of Health; NOT: nocturnal oxygen therapy; CPAP: continuous positive airway pressure; AHI: apnoea–hypopnoea index; NHLBI: National Heart, Lung, and Blood Institute; UCSD: University of California San Diego; HLSE: healthy lifestyle and sleep education; ATS: American Thoracic Society; NIHR: National Institute for Health and Care Research; AHA: American Heart Association; ODI: oxygen desaturation index; RDI: respiratory disturbance index. ^#^: per-protocol data; ^¶^: intention-to-treat data; ^+^: AHI value following the surgery but prior to the intervention with sham or NOT.

Oxygen flow rate, duration of intervention and outcome measures varied among the studies. Details of the included studies can be found in table 2.

### NOT effects on AHI and ODI

Seven studies compared AHI in oxygen *versus* sham groups. The definition of hypopnoea varied among studies. Some studies [15, 18, 19] required ≥30% reduction in airflow in accordance with 2012 American Academy of Sleep Medicine (AASM) criteria [21] whereas other studies [11, 12, 14, 16] used ≥50% reduction in airflow criterion for scoring hypopnoeas. All studies reported a standard AHI that includes a desaturation criterion to score hypopnoeas. Four studies [15, 16, 18, 19] calculated AHI without the desaturation requirement, *i.e.* using flow-based criteria alone.

**FIGURE 2:**
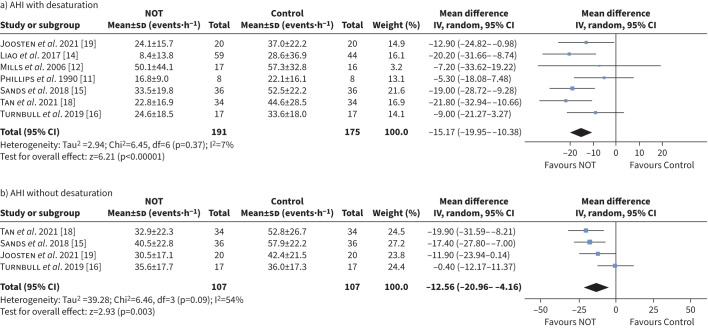
Forest plot of apnoea–hypopnoea index (AHI) a) with desaturation and b) without desaturation. NOT: nocturnal oxygen therapy; IV: inverse variance.

**FIGURE 3:**
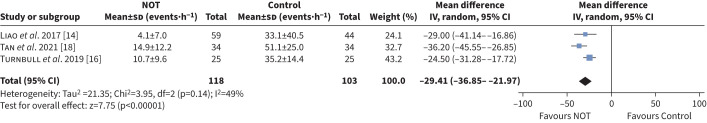
Forest plot of oxygen desaturation index. NOT: nocturnal oxygen therapy; IV: inverse variance.

Four studies [14, 15, 18, 19] showed that NOT significantly reduced AHI compared to the control whereas three [11, 12, 16] found no significant difference between the two groups. Meta-analysis of the data showed significant reductions in both types of AHI: standard AHI (mean difference (MD) −15.17 events·h^−1^, 95% CI −19.95– −10.38 events·h^−1^, p<0.00001, I^2^=7%, figure 2a) and flow-based AHI (MD −12.56 events·h^−1^, 95% CI −20.96–4.16 events·h^−1^, p=0.003, I^2^= 54%, figure 2b). As expected, NOT significantly reduced the ODI compared to control (MD −29.41 events·h^−1^, 95% CI −36.85– −21.97 events·h^−1^, p*<*0.00001, I^2^= 49%, figure 3).

Given the variability in study design, we performed subgroup analyses of standard AHI depending on oxygen flow rate (low flow 2–3 L·min^−1^ *versus* higher flow >3 L·min^−^^1^), duration of NOT (1 night *versus* longer) and types of study (randomised crossover studies *versus* RCTs). All these subgroup analyses showed significant reductions in AHI (supplementary figure S2).

### NOT effects on arousal index and apnoea duration

As shown in supplementary figure S1, NOT did not significantly reduce the arousal index (MD −5.72 events·h^−1^, 95% CI −12.81–1.36 events·h^−1^, p=0.110, I^2^=75%). Apnoea duration was reported in only two of the studies and we have therefore not performed a meta-analysis for this outcome. One study showed that oxygen therapy prolonged the mean apnoea duration and another study showed no difference between the two groups [14].

### The effect of NOT on blood pressure

**FIGURE 4:**
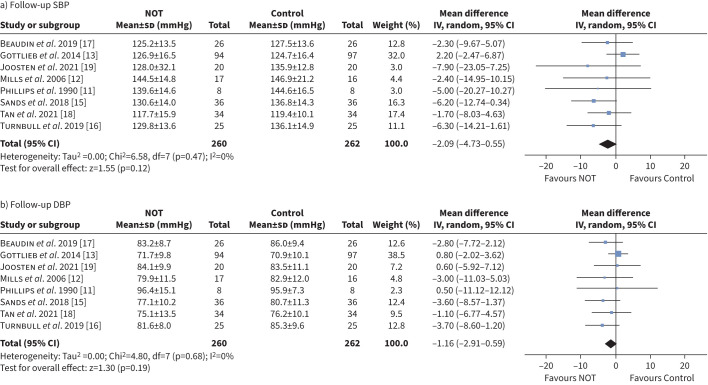
Forest plot of follow-up a) systolic blood pressure (SBP) and b) diastolic blood pressure (DBP). NOT: nocturnal oxygen therapy; IV: inverse variance.

Eight studies reported post-intervention blood pressure (figure 4) [11–13, 15–19]. Both office and home blood pressure measurements were reported in one study [16], another study reported ambulatory blood pressure and 24-h mean blood pressure measurement [13], and the remaining studies reported office blood pressure only. Change in blood pressure following intervention was reported in two studies [15, 16] and calculated for four more studies [11–13, 17] from mean differences and pooled sds.

NOT had no significant effect on either follow-up SBP (MD −2.09 mmHg, 95% CI −4.73–0.55 mmHg, p*=*0.120, I^2^=0%) or DBP (MD −1.16 mmHg, 95% CI −2.91–0.59 mmHg, p*=*0.190, I^2^=0%) (figure 4).

Given the variability in study design, we performed subgroup analysis depending on oxygen flow rate (low flow 2–3 L·min^−1^ *versus* higher flow >3 L·min^−1^), duration of NOT (1 night *versus* longer) and types of study (randomised crossover studies *versus* RCTs). Subgroup analyses of crossover studies in which the same subjects received the intervention and the sham (so the baseline values were identical for both arms) revealed lower follow-up SBP after NOT (MD −4.71 mmHg, 95% CI −8.42– −1.01 mmHg, p*=*0.010, I^2^=0%) but not DBP (supplementary figures S3 and S4). There were no significant differences for either SBP or DBP in RCTs. In studies using an oxygen flow rate >3 L·min^−1^, NOT had a significant effect on SBP (MD −5.18 mmHg, 95% CI −9.06– −1.30 mmHg, p=0.009, I^2^=0%) and DBP (MD −2.58 mmHg, 95% CI −5.12– −0.03 mmHg, p=0.050, I^2^=0%), while there was no effect with lower oxygen flow rates. There was no significant difference in the effect of NOT on either SBP or DBP in studies with 1-night duration or longer duration of intervention.

**FIGURE 5:**
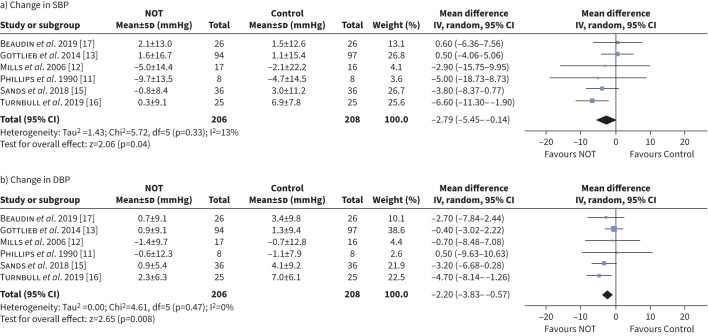
Forest plot of change in a) systolic blood pressure (SBP) and b) diastolic blood pressure (DBP). NOT: nocturnal oxygen therapy; IV: inverse variance.

Contrary to the follow-up blood pressure data, meta-analysis of mean changes in blood pressure showed statistically significant reductions in NOT groups for SBP (MD −2.79 mmHg, 95% CI −5.45– −0.14 mmHg, p*=*0.040, I^2^=13%) and DBP (MD −2.20 mmHg, 95% CI −3.83– −0.57 mmHg, p*=*0.008, I^2^=0%) (figure 5). Subgroup analyses for the change in SBP and DBP were also performed (supplementary figures S5 and S6).

### NOT effects on heart rate

Five studies [12, 15–18] had follow-up heart rate data and change in heart rate data was calculable in four of them. NOT had no statistically significant effect on either follow-up heart rate or heart rate change (MD −0.86 bpm, 95% CI −3.45–1.74 bpm, p=0.520, I^2^=30% and MD 0.24 bpm, 95% CI −2.08–2.56 bpm, p=0.840, I^2^=0%, respectively) (supplementary figures S7 and S8).

### GRADE quality of evidence assessment

For our main outcomes, we assessed quality of evidence using Grading of Recommendations, Assessment, Development and Evaluation (GRADE) [22]. Funnel plots for risk of publication bias assessment can be found in supplementary figures S9–S16. The final GRADE assessment can be seen in table 3.

**TABLE 3:** GRADE quality of evidence assessment for important outcomes

**Outcome**	**Number of participants (studies)**	**GRADE assessment**
**AHI with desaturation**	251 participants (7 studies)	⊕⊕⊕Ο Moderate Reduced by one for imprecision
**AHI without desaturation**	107 participants (4 studies)	⊕⊕ΟΟ Low Reduced by one for inconsistency Reduced by one for imprecision
**ODI**	162 participants (3 studies)	⊕⊕ΟΟ Low Reduced by one for inconsistency Reduced by one for imprecision
**Arousal index**	193 participants (4 studies)	⊕ΟΟΟ Very Low Reduced by one for inconsistency Reduced by one for imprecision Reduced by one for risk of publication bias
**Follow-up SBP**	399 participants (8 studies)	⊕⊕ΟΟ Low Reduced by one for inconsistency Reduced by one for risk of publication bias
**Follow-up DBP**	399 participants (8 studies)	⊕⊕⊕Ο Moderate Reduced by one for inconsistency
**Change in SBP**	345 participants (6 studies)	⊕⊕ΟΟ Low Reduced by one for inconsistency Reduced by one for imprecision
**Change in DBP**	345 participants (6 studies)	⊕⊕ΟΟ Low Reduced by one for inconsistency Reduced by one for imprecision

GRADE: Grading of Recommendations, Assessment, Development and Evaluations; AHI: apnoea–hypopnoea index; ODI: oxygen desaturation index; SBP: systolic blood pressure; DBP: diastolic blood pressure.

## Discussion

Our systematic review and meta-analysis shows that NOT therapy reduced the AHI and may have led to small reductions in blood pressure, at least in the short term. The magnitude of the reduction in AHI was dependent on whether the AHI was scored using standard techniques including oxygen desaturation or using flow-based techniques without a desaturation criterion. NOT therapy had no effect on blood pressure at follow-up, but did reduce blood pressure when assessing a change in blood pressure from baseline. This review expands on previous meta-analyses of the effect of NOT on OSA and is novel in reporting the effect of NOT on blood pressure compared to sham/control.

The current gold standard treatment for OSA is CPAP therapy. CPAP is effective in abolishing intermittent hypoxia secondary to upper airway collapse, and has been shown to reduce AHI and arousals and improve blood pressure [23, 24]. The main limiting factor for CPAP treatment is poor tolerance; CPAP nonadherence rates have been consistently as high as 34% despite advances in machine dynamics and behavioural interventions [25]. This has necessitated searches for alternative treatment options. Mandibular advancement devices, positional modifiers and surgical treatment are possible second-line options for OSA patients [26]. NOT has also long been considered as an alternative therapy for OSA patients owing to its attenuating effects on intermittent hypoxia; however, its effects on AHI and blood pressure are more controversial.

A meta-analysis by Mehta *et al*. [7] found that NOT showed similar improvements in nocturnal oxygen saturations compared to CPAP but CPAP led to a significant reduction in AHI compared to NOT. They also found reductions in sleep disordered breathing with oxygen compared to air in observational studies but not in randomised studies comparing NOT to placebo CPAP. This is in contrast to our study, which was able to include more randomised studies and showed reductions in AHI in NOT groups.

NOT can affect the measurement of hypopnoeas when using standard criteria and our analysis highlights the need for standardised flow-based AHI measurements when assessing the effects of NOT. Studies included in this meta-analysis used different methods to score the AHI and there is no consensus for how the AHI should be reported when using NOT therapy (reference AASM guidelines [21]). In our opinion, flow-based AHI measurements without using oxygen desaturations should be used to score hypopnoeas in studies assessing NOT.

OSA is a risk factor for hypertension and hypertension-related target organ damage [27]. Cardiovascular risks predisposed by high blood pressure are one reason for treatment of OSA [28]. CPAP has a modest effect on blood pressure [29], but the effects of NOT are not clear.

In accordance with our results, a subgroup analysis in another meta-analysis comparing the effects of CPAP and NOT on blood pressure showed that higher oxygen flow rate (≥4 L·min^−1^) enhanced the effects of oxygen on blood pressure more than lower flow rates [30].

Most studies recorded blood pressure using single-visit office measurements. Guidelines recommend against this approach for the diagnosis of hypertension [31], and we suggest using either ambulatory or home blood pressure measurements at a standardised time of day to monitor the effect of NOT therapy in OSA. There were insufficient studies to perform a meta-analysis for subgroups based on home or ambulatory blood pressure recordings.

Not all studies allowed for baseline blood pressure when assessing the follow-up blood pressure. In our study, there was no significant difference in either SBP or DBP when only considering follow-up values. Furthermore, when considering the subgroup of crossover studies, in which individuals are likely to have similar baseline blood pressure values in both arms, there was a significant change in SBP but not DBP. We would suggest that future studies also include methodology to allow for baseline blood pressure values when considering blood pressure as an outcome.

The exact mechanism by which NOT lowers the AHI and blood pressure is not known. There has been increased recognition that there are many nonanatomic contributors to the pathogenesis of OSA, including high loop gain and low arousal threshold [32]. Approximately one in three patients with OSA have an elevated loop gain [32]. Supplemental oxygen therapy can reduce loop gain and has the potential to reduce the severity of OSA [33]. While our meta-analysis cannot reveal the underlying mechanism of reduction in AHI, it did show a large effect of NOT on the AHI. This suggests that improvements in the AHI may not be restricted to individuals with higher loop gain.

The underlying mechanism by which NOT might reduce blood pressure is not known. Animal and human experimental models suggest a central role of intermittent hypoxia and sympathetic activation in elevations in blood pressure in OSA [34, 35]. NOT may affect blood pressure by reducing intermittent hypoxia without abolishing all obstructive events. The extent of hypoxic burden related to sleep apnoea has been shown to be associated with cardiovascular risks. It may be that the extent of hypoxia related to sleep apnoea influences the effect of NOT on blood pressure but these data were not routinely collected within the included studies.

Our meta-analysis has several limitations. First, the total numbers of included studies and participants were relatively low. Second, the study population had wide heterogeneity with different OSA severity and it was not possible to assess the NOT effects on OSA patients of each severity group. Third, the duration of follow-up in the studies was relatively short, with the majority of studies lasting ≤2 weeks and only two studies lasting >2 weeks, which limits assessment of long-term effects of NOT. Additionally, most of the studies reported mean baseline and follow-up data separately, and mean pre- and post-intervention changes and sds were calculated from the pooled sd formula, which may be different from the mean change value of the study raw datasets. Furthermore, as described above, AHI and blood pressure measurements were not standardised across studies, which may affect outcomes. The AHI was measured using flow from nasal cannulae with the concomitant administration of NOT with either a facemask or nasal cannula interface. Reassuringly, crossover studies in which NOT was compared to sham oxygen at similar flow rates found comparable reductions in the AHI to other studies, suggesting that this did not bias the results (supplementary figure S2a).

In conclusion, this meta-analysis suggests that NOT reduced both the AHI and the extent of intermittent hypoxia. These data highlight the possible beneficial effects of NOT therapy in OSA. However, included studies differed in terms of length of follow-up, study design, oxygen flow rates, scoring classifications for the AHI and the method of blood pressure recordings. Further work is needed to determine the optimal flow rate and the longer-term efficacy and tolerability of oxygen therapy. Caution needs to be exercised because NOT has the potential to prolong apnoea duration and cause hypercapnia in high-risk individuals. The effects of NOT on blood pressure were varied and more work is needed. Our study highlights the need for standardised methods for flow-based AHI and blood pressure measurement and adjustment for baseline blood pressure values.

## Supplementary material

10.1183/16000617.0173-2023.Supp1 **Please note:** supplementary material is not edited by the Editorial Office, and is uploaded as it has been supplied by the author.
Supplementary material ERR-0173-2023.SUPPLEMENT
