# Supplementary material for: Nocturnal oxygen therapy in obstructive sleep apnoea: a systematic review and meta-analysis
Source: Eur Respir Rev. 2024 Mar 20;33(171):230173. doi: 10.1183/16000617.0173-2023 (PMC10951857; doi:10.1183/16000617.0173-2023)

Nocturnal Oxygen Therapy in Obstructive Sleep Apnoea: Systematic Review and Meta-analysis

Search strategies designed and carried out on **11/04/2022** and updated in full on **14/04/2023**.

Search Results

	11/04/2022 results	14/04/2023 results
PubMed	503	525
Ovid Embase	972	1066
Cochrane CENTRAL	126	135
Cochrane Database of Systematic Reviews	0	0
Scopus	293	280
Web of Science – Core Collection	254	276
Total	2148	2282
Total after deduplication	1194	
Unique since 11/04/2022		101

Search Strategies

PubMed

((("Sleep Apnea, Obstructive"[Mesh]) OR ("obstructive sleep apnea*"[Title/Abstract] OR "obstructive sleep apnoea*"[Title/Abstract] OR OSA[Title/Abstract] OR OSAHS[Title/Abstract])) AND (("Oxygen Inhalation Therapy"[Mesh]) OR ("supplemental oxygen"[Title/Abstract] OR "nocturnal oxygen"[Title/Abstract]))) *Filters applied: English.*

Database: Embase 1974 to present

Search Strategy:

----

1 exp sleep disordered breathing/ (70913)

2 "obstructive sleep apn?ea*".ti,ab. (57941)

3 (OSA or OSAHS).ti,ab. (38420)

4 1 or 2 or 3 (93318)

5 nocturnal oxygen therapy/ (70)

6 ("nocturnal oxygen" or "supplemental oxygen" or "night oxygen therapy" or "nocturnal O2").ti,ab. (10296)

7 5 or 6 (10332)

8 4 and 7 (1108)

9 8 (1108)

10 limit 9 to english language (1066)

Cochrane Central Register of Controlled Trials

Issue 4 of 12, April 2023

Cochrane Database of Systematic Reviews

Issue 4 of 12, April 2023

#1 MeSH descriptor: [Sleep Apnea, Obstructive] explode all trees 2616

#2 ("obstructive sleep apnea*" OR "obstructive sleep apnoea*" OR OSA OR OSAHS):ti,ab,kw 6541

#3 #1 or #2 6857

#4 MeSH descriptor: [Oxygen Inhalation Therapy] explode all trees 2017

#5 ("nocturnal oxygen" or "supplemental oxygen" or "night oxygen therapy" or "nocturnal O2"):ti,ab,kw 2377

#6 #4 or #5 4074

#7 #3 and #6 135

Scopus

(TITLE-ABS-KEY ("obstructive sleep apn?ea*" OR osa OR osahs) AND TITLE-ABS-KEY ("nocturnal oxygen" OR "supplemental oxygen" OR "night oxygen therapy" OR "nocturnal O2")) AND (LIMIT-TO (LANGUAGE , "English"))

Web of Science – Core Collection

#1 TOPIC: "obstructive sleep apn?ea*" OR osa OR osahs

#2 TOPIC: "nocturnal oxygen" OR "supplemental oxygen" OR "night oxygen therapy" OR "nocturnal O2"

#3 #1 AND #2 Refined by: Languages - English

Google Scholar search strings – screen the first 100 results for additional relevant references once you've completed the initial screening of the results from the bibliographic database searches

("obstructive sleep apnoea"|"obstructive sleep apnea"|OSA|OSAHS)("nocturnal oxygen"|"supplemental oxygen"|"night oxygen therapy"|"nocturnal O2")

https://scholar.google.co.uk/scholar?hl=en&as_sdt=0%2C5&q=%28%22obstructive+sleep+apnoea%22%7C%22obstructive+sleep+apnea%22%7COSA%7COSAHS%29%28%22nocturnal+oxygen%22%7C+%22supplemental+oxygen%22%7C%22night+oxygen+therapy%22%7C%22nocturnal+O2%22%29&btnG=

Supplementary Figure S1: Effect on arousal index

Study or Subgroup	NOT Mean	NOT SD	NOT Total	Control Mean	Control SD	Control Total	Weight	Mean Difference IV, Random, 95% CI
Joosten 2021	33	15.3	20	41.1	19.5	20	19.3%	-8.10 [-18.96, 2.76]
Liao 2017	9.5	6.6	59	8.5	7.8	44	33.4%	1.00 [-1.85, 3.85]
Sands 2018	35.9	20.4	36	50.3	22.2	36	21.0%	-14.40 [-24.25, -4.55]
Tan 2021	22.7	13.5	34	28.3	15.9	34	26.3%	-5.60 [-12.61, 1.41]
**Total (95% CI)**			**149**			**134**	**100.0%**	**-5.72 [-12.81, 1.36]**

Heterogeneity: Tau² = 36.93; Chi² = 12.24, df = 3 (P = 0.007); I² = 75%
Test for overall effect: Z = 1.58 (P = 0.11)

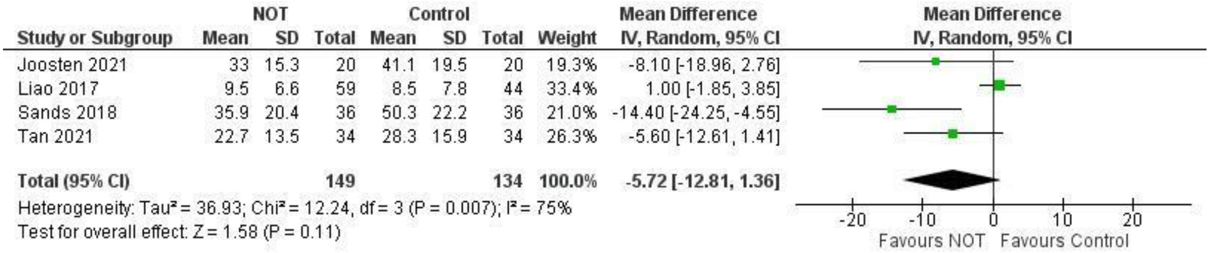

Supplementary Figure S2: Subgroup analyses for standard AHI

a) Based on study type

Study or Subgroup	NOT Mean	NOT SD	NOT Total	Control Mean	Control SD	Control Total	Weight	Mean Difference IV, Random, 95% CI
**Crossover Studies**								
Joosten 2021	24.1	15.7	20	37	22.2	20	14.9%	−12.90 [−24.82, −0.98]
Phillips 1990	16.8	9	8	22.1	16.1	8	13.1%	−5.30 [−18.08, 7.48]
Sands 2018	33.5	19.8	36	52.5	22.2	36	21.6%	−19.00 [−28.72, −9.28]
Tan 2021	22.8	16.9	34	44.6	28.5	34	16.9%	−21.80 [−32.94, −10.66]
Turnbull 2019	24.6	18.5	17	33.6	18	17	14.1%	−9.00 [−21.27, 3.27]
**Subtotal (95% CI)**			**115**			**115**	**80.7%**	**−14.32 [−20.22, −8.42]**
Heterogeneity: $Tau^2 = 11.16$; $Chi^2 = 5.31$, df = 4 (P = 0.26); $I^2 = 25\%$								
Test for overall effect: Z = 4.75 (P < 0.00001)								
**RCT**								
Liao 2017	8.4	13.8	59	28.6	36.9	44	16.1%	−20.20 [−31.66, −8.74]
Mills 2006	50.1	44.1	17	57.3	32.8	16	3.2%	−7.20 [−33.62, 19.22]
**Subtotal (95% CI)**			**76**			**60**	**19.3%**	**−18.14 [−28.65, −7.63]**
Heterogeneity: $Tau^2 = 0.00$; $Chi^2 = 0.78$, df = 1 (P = 0.38); $I^2 = 0\%$								
Test for overall effect: Z = 3.38 (P = 0.0007)								
**Total (95% CI)**			**191**			**175**	**100.0%**	**−15.17 [−19.95, −10.38]**
Heterogeneity: $Tau^2 = 2.94$; $Chi^2 = 6.45$, df = 6 (P = 0.37); $I^2 = 7\%$								
Test for overall effect: Z = 6.21 (P < 0.00001)								
Test for subgroup differences: $Chi^2 = 0.39$, df = 1 (P = 0.53), $I^2 = 0\%$								

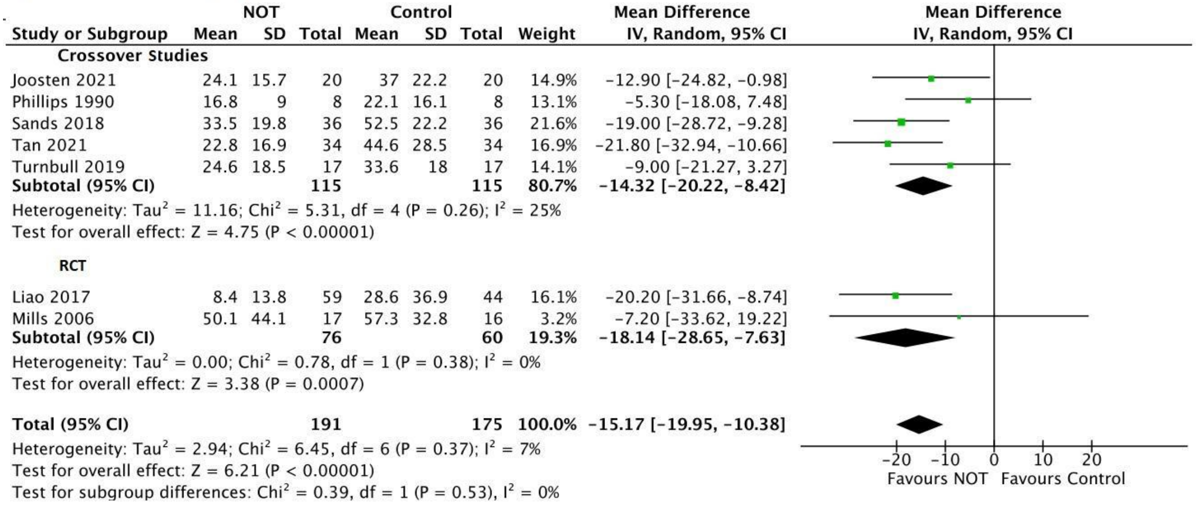

b)Based on oxygen flow rate

Study or Subgroup	NOT Mean	NOT SD	NOT Total	Control Mean	Control SD	Control Total	Weight	Mean Difference IV, Random, 95% CI
**2–3L/min**								
Liao 2017	8.4	13.8	59	28.6	36.9	44	16.1%	−20.20 [−31.66, −8.74]
Mills 2006	50.1	44.1	17	57.3	32.8	16	3.2%	−7.20 [−33.62, 19.22]
Tan 2021	22.8	16.9	34	44.6	28.5	34	16.9%	−21.80 [−32.94, −10.66]
**Subtotal (95% CI)**			**110**			**94**	**36.2%**	**−19.87 [−27.51, −12.22]**
Heterogeneity: $Tau^2 = 0.00$; $Chi^2 = 1.00$, df = 2 (P = 0.61); $I^2 = 0\%$								
Test for overall effect: Z = 5.09 (P < 0.00001)								
**More than 3L/min**								
Joosten 2021	24.1	15.7	20	37	22.2	20	14.9%	−12.90 [−24.82, −0.98]
Phillips 1990	16.8	9	8	22.1	16.1	8	13.1%	−5.30 [−18.08, 7.48]
Sands 2018	33.5	19.8	36	52.5	22.2	36	21.6%	−19.00 [−28.72, −9.28]
Turnbull 2019	24.6	18.5	17	33.6	18	17	14.1%	−9.00 [−21.27, 3.27]
**Subtotal (95% CI)**			**81**			**81**	**63.8%**	**−12.54 [−18.53, −6.56]**
Heterogeneity: $Tau^2 = 2.93$; $Chi^2 = 3.25$, df = 3 (P = 0.35); $I^2 = 8\%$								
Test for overall effect: Z = 4.11 (P < 0.0001)								
**Total (95% CI)**			**191**			**175**	**100.0%**	**−15.17 [−19.95, −10.38]**
Heterogeneity: $Tau^2 = 2.94$; $Chi^2 = 6.45$, df = 6 (P = 0.37); $I^2 = 7\%$								
Test for overall effect: Z = 6.21 (P < 0.00001)								
Test for subgroup differences: $Chi^2 = 2.18$, df = 1 (P = 0.14), $I^2 = 54.2\%$								

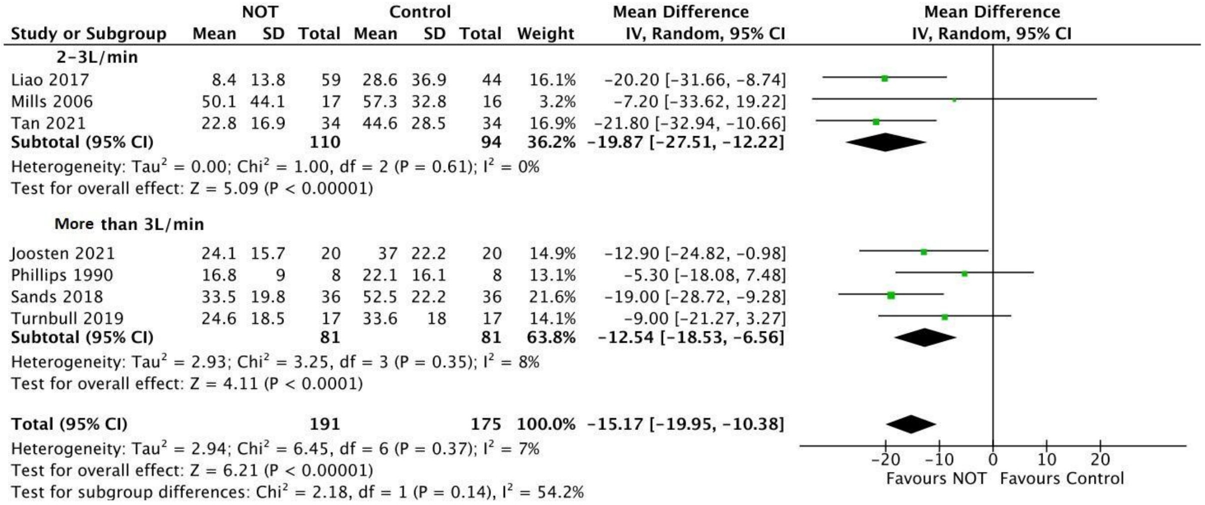

c) Based on duration of intervention

Study or Subgroup	NOT Mean	NOT SD	NOT Total	Control Mean	Control SD	Control Total	Weight	Mean Difference IV, Random, 95% CI
**1 night**								
Joosten 2021	24.1	15.7	20	37	22.2	20	14.9%	−12.90 [−24.82, −0.98]
Sands 2018	33.5	19.8	36	52.5	22.2	36	21.6%	−19.00 [−28.72, −9.28]
Tan 2021	22.8	16.9	34	44.6	28.5	34	16.9%	−21.80 [−32.94, −10.66]
**Subtotal (95% CI)**			**90**			**90**	**53.5%**	**−18.21 [−24.45, −11.97]**
Heterogeneity: $Tau^2 = 0.00$; $Chi^2 = 1.19$, df = 2 (P = 0.55); $I^2 = 0\%$								
Test for overall effect: Z = 5.72 (P < 0.00001)								
**More than 1 night**								
Liao 2017	8.4	13.8	59	28.6	36.9	44	16.1%	−20.20 [−31.66, −8.74]
Mills 2006	50.1	44.1	17	57.3	32.8	16	3.2%	−7.20 [−33.62, 19.22]
Phillips 1990	16.8	9	8	22.1	16.1	8	13.1%	−5.30 [−18.08, 7.48]
Turnbull 2019	24.6	18.5	17	33.6	18	17	14.1%	−9.00 [−21.27, 3.27]
**Subtotal (95% CI)**			**101**			**85**	**46.5%**	**−11.64 [−18.92, −4.35]**
Heterogeneity: $Tau^2 = 6.37$; $Chi^2 = 3.37$, df = 3 (P = 0.34); $I^2 = 11\%$								
Test for overall effect: Z = 3.13 (P = 0.002)								
**Total (95% CI)**			**191**			**175**	**100.0%**	**−15.17 [−19.95, −10.38]**
Heterogeneity: $Tau^2 = 2.94$; $Chi^2 = 6.45$, df = 6 (P = 0.37); $I^2 = 7\%$								
Test for overall effect: Z = 6.21 (P < 0.00001)								
Test for subgroup differences: $Chi^2 = 1.80$, df = 1 (P = 0.18), $I^2 = 44.5\%$								

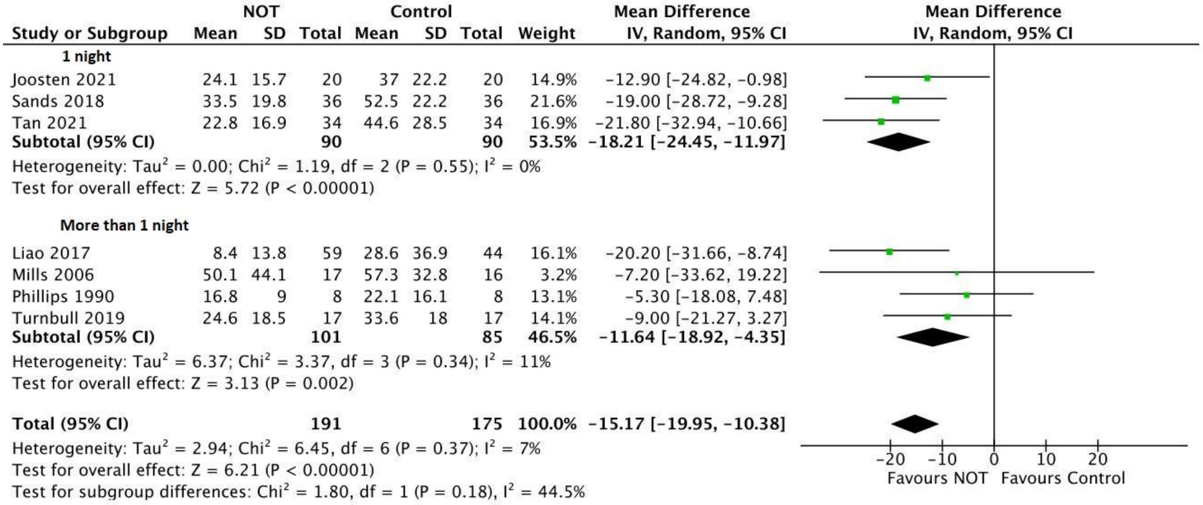

Supplementary Figure S3: Subgroup analyses for follow-up SBP

a) Based on study type

Study or Subgroup	NOT Mean	NOT SD	NOT Total	Control Mean	Control SD	Control Total	Weight	Mean Difference IV, Random, 95% CI
**Crossover Studies**								
Joosten 2021	128	32.1	20	135.9	12.8	20	3.0%	-7.90 [-23.05, 7.25]
Phillips 1990	139.6	14.6	8	144.6	16.5	8	3.0%	-5.00 [-20.27, 10.27]
Sands 2018	130.6	14	36	136.8	14.3	36	16.3%	-6.20 [-12.74, 0.34]
Tan 2021	117.7	15.9	34	119.4	10.1	34	17.4%	-1.70 [-8.03, 4.63]
Turnbull 2019	129.8	13.6	25	136.1	14.9	25	11.1%	-6.30 [-14.21, 1.61]
**Subtotal (95% CI)**			**123**			**123**	**50.8%**	**-4.71 [-8.42, -1.01]**
Heterogeneity: Tau² = 0.00; Chi² = 1.39, df = 4 (P = 0.85); I² = 0%								
Test for overall effect: Z = 2.50 (P = 0.01)								
**RCT**								
Beaudin 2019	125.2	13.5	26	127.5	13.6	26	12.8%	-2.30 [-9.67, 5.07]
Gottlieb 2014	126.9	16.5	94	124.7	16.4	97	32.0%	2.20 [-2.47, 6.87]
Mills 2006	144.5	14.8	17	146.9	21.2	16	4.4%	-2.40 [-14.95, 10.15]
**Subtotal (95% CI)**			**137**			**139**	**49.2%**	**0.61 [-3.15, 4.37]**
Heterogeneity: Tau² = 0.00; Chi² = 1.27, df = 2 (P = 0.53); I² = 0%								
Test for overall effect: Z = 0.32 (P = 0.75)								
**Total (95% CI)**			**260**			**262**	**100.0%**	**-2.09 [-4.73, 0.55]**
Heterogeneity: Tau² = 0.00; Chi² = 6.58, df = 7 (P = 0.47); I² = 0%								
Test for overall effect: Z = 1.55 (P = 0.12)								
Test for subgroup differences: Chi² = 3.92, df = 1 (P = 0.05), I² = 74.5%								

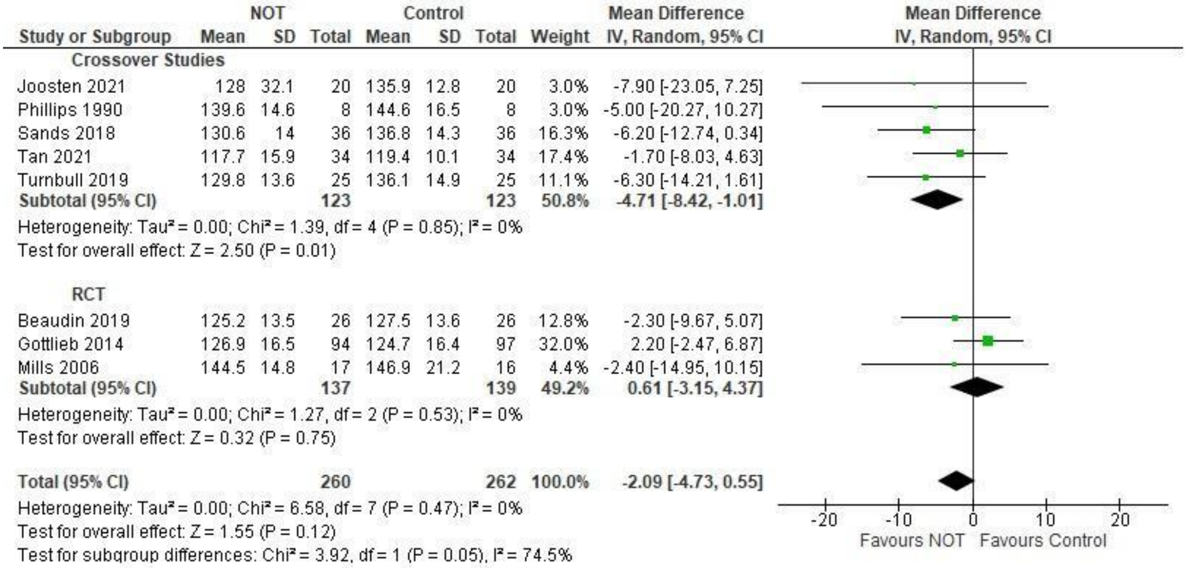

b) Based on oxygen flow rate

Study or Subgroup	NOT Mean	NOT SD	NOT Total	Control Mean	Control SD	Control Total	Weight	Mean Difference IV, Random, 95% CI
**2-3 L/min**								
Gottlieb 2014	126.9	16.5	94	124.7	16.4	97	32.0%	2.20 [-2.47, 6.87]
Mills 2006	144.5	14.8	17	146.9	21.2	16	4.4%	-2.40 [-14.95, 10.15]
Tan 2021	117.7	15.9	34	119.4	10.1	34	17.4%	-1.70 [-8.03, 4.63]
**Subtotal (95% CI)**			**145**			**147**	**53.7%**	**0.56 [-3.04, 4.16]**
Heterogeneity: Tau² = 0.00; Chi² = 1.18, df = 2 (P = 0.55); I² = 0%								
Test for overall effect: Z = 0.31 (P = 0.76)								
**More than 3 L/min**								
Beaudin 2019	125.2	13.5	26	127.5	13.6	26	12.8%	-2.30 [-9.67, 5.07]
Joosten 2021	128	32.1	20	135.9	12.8	20	3.0%	-7.90 [-23.05, 7.25]
Phillips 1990	139.6	14.6	8	144.6	16.5	8	3.0%	-5.00 [-20.27, 10.27]
Sands 2018	130.6	14	36	136.8	14.3	36	16.3%	-6.20 [-12.74, 0.34]
Turnbull 2019	129.8	13.6	25	136.1	14.9	25	11.1%	-6.30 [-14.21, 1.61]
**Subtotal (95% CI)**			**115**			**115**	**46.3%**	**-5.18 [-9.06, -1.30]**
Heterogeneity: Tau² = 0.00; Chi² = 0.88, df = 4 (P = 0.93); I² = 0%								
Test for overall effect: Z = 2.62 (P = 0.009)								
**Total (95% CI)**			**260**			**262**	**100.0%**	**-2.09 [-4.73, 0.55]**
Heterogeneity: Tau² = 0.00; Chi² = 6.58, df = 7 (P = 0.47); I² = 0%								
Test for overall effect: Z = 1.55 (P = 0.12)								
Test for subgroup differences: Chi² = 4.52, df = 1 (P = 0.03), I² = 77.9%								

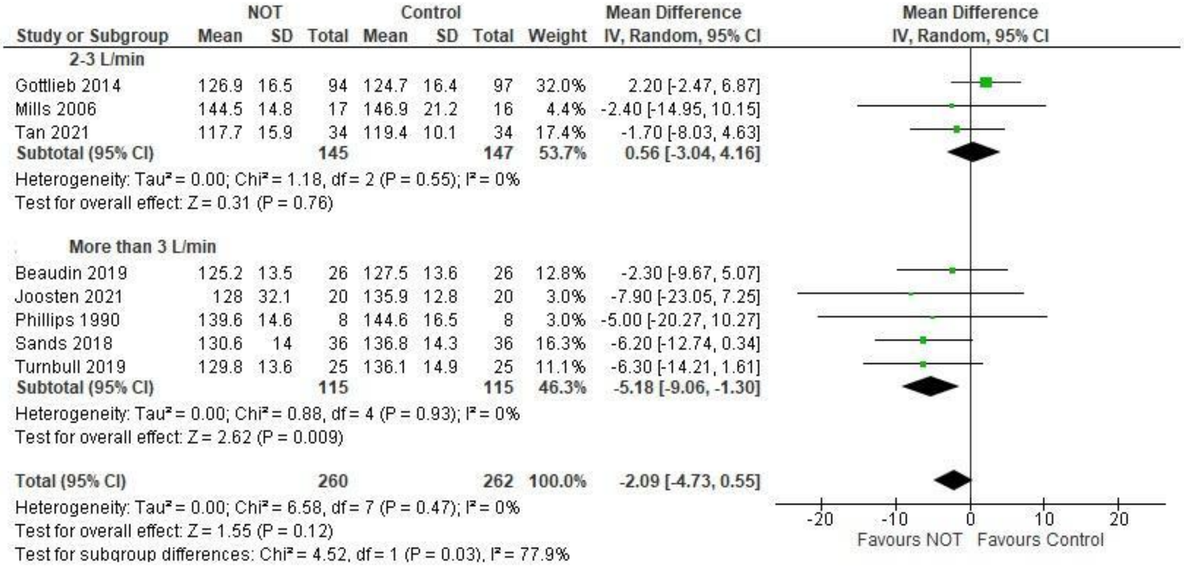

c) Based on duration of intervention

Study or Subgroup	NOT Mean	NOT SD	NOT Total	Control Mean	Control SD	Control Total	Weight	Mean Difference IV, Random, 95% CI
**1 night**								
Joosten 2021	128	32.1	20	135.9	12.8	20	3.0%	-7.90 [-23.05, 7.25]
Sands 2018	130.6	14	36	136.8	14.3	36	16.3%	-6.20 [-12.74, 0.34]
Tan 2021	117.7	15.9	34	119.4	10.1	34	17.4%	-1.70 [-8.03, 4.63]
**Subtotal (95% CI)**			**90**			**90**	**36.7%**	**-4.21 [-8.57, 0.15]**
Heterogeneity: Tau² = 0.00; Chi² = 1.19, df = 2 (P = 0.55); I² = 0%								
Test for overall effect: Z = 1.89 (P = 0.06)								
**More than 1 night**								
Beaudin 2019	125.2	13.5	26	127.5	13.6	26	12.8%	-2.30 [-9.67, 5.07]
Gottlieb 2014	126.9	16.5	94	124.7	16.4	97	32.0%	2.20 [-2.47, 6.87]
Mills 2006	144.5	14.8	17	146.9	21.2	16	4.4%	-2.40 [-14.95, 10.15]
Phillips 1990	139.6	14.6	8	144.6	16.5	8	3.0%	-5.00 [-20.27, 10.27]
Turnbull 2019	129.8	13.6	25	136.1	14.9	25	11.1%	-6.30 [-14.21, 1.61]
**Subtotal (95% CI)**			**170**			**172**	**63.3%**	**-0.87 [-4.18, 2.45]**
Heterogeneity: Tau² = 0.00; Chi² = 3.96, df = 4 (P = 0.41); I² = 0%								
Test for overall effect: Z = 0.51 (P = 0.61)								
**Total (95% CI)**			**260**			**262**	**100.0%**	**-2.09 [-4.73, 0.55]**
Heterogeneity: Tau² = 0.00; Chi² = 6.58, df = 7 (P = 0.47); I² = 0%								
Test for overall effect: Z = 1.55 (P = 0.12)								
Test for subgroup differences: Chi² = 1.43, df = 1 (P = 0.23), I² = 30.3%								

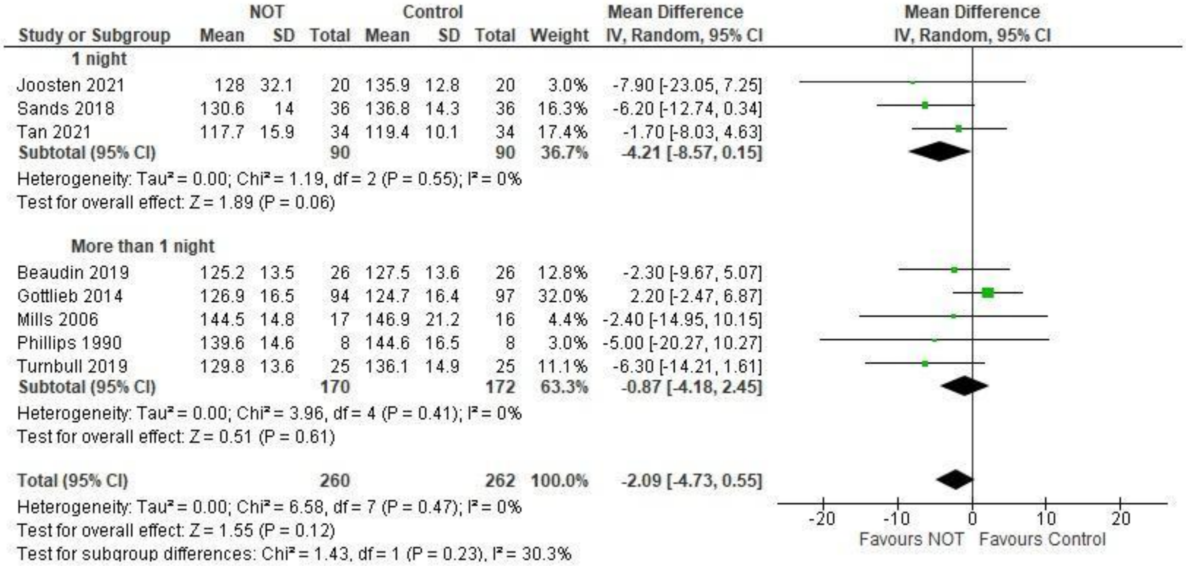

Supplementary Figure S4: Subgroup analysis for follow-up DBP

a) Based on study type

Study or Subgroup	NOT Mean	NOT SD	NOT Total	Control Mean	Control SD	Control Total	Weight	Mean Difference IV, Random, 95% CI
**Crossover Studies**								
Joosten 2021	84.1	9.9	20	83.5	11.1	20	7.2%	0.60 [-5.92, 7.12]
Phillips 1990	96.4	15.1	8	95.9	7.3	8	2.3%	0.50 [-11.12, 12.12]
Sands 2018	77.1	10.2	36	80.7	11.3	36	12.4%	-3.60 [-8.57, 1.37]
Tan 2021	75.1	13.5	34	76.2	10.1	34	9.5%	-1.10 [-6.77, 4.57]
Turnbull 2019	81.6	8	25	85.3	9.6	25	12.8%	-3.70 [-8.60, 1.20]
**Subtotal (95% CI)**			**123**			**123**	**44.2%**	**-2.19 [-4.83, 0.44]**
Heterogeneity: Tau² = 0.00; Chi² = 1.73, df = 4 (P = 0.79); I² = 0%								
Test for overall effect: Z = 1.63 (P = 0.10)								
**RCT**								
Beaudin 2019	83.2	8.7	26	86	9.4	26	12.6%	-2.80 [-7.72, 2.12]
Gottlieb 2014	71.7	9.8	94	70.9	10.1	97	38.5%	0.80 [-2.02, 3.62]
Mills 2006	79.9	11.5	17	82.9	12	16	4.8%	-3.00 [-11.03, 5.03]
**Subtotal (95% CI)**			**137**			**139**	**55.8%**	**-0.34 [-2.70, 2.01]**
Heterogeneity: Tau² = 0.02; Chi² = 2.01, df = 2 (P = 0.37); I² = 0%								
Test for overall effect: Z = 0.29 (P = 0.77)								
**Total (95% CI)**			**260**			**262**	**100.0%**	**-1.16 [-2.91, 0.59]**

Heterogeneity: Tau² = 0.00; Chi² = 4.80, df = 7 (P = 0.68); I² = 0%
Test for overall effect: Z = 1.30 (P = 0.19)
Test for subgroup differences: Chi² = 1.05, df = 1 (P = 0.30), I² = 5.0%

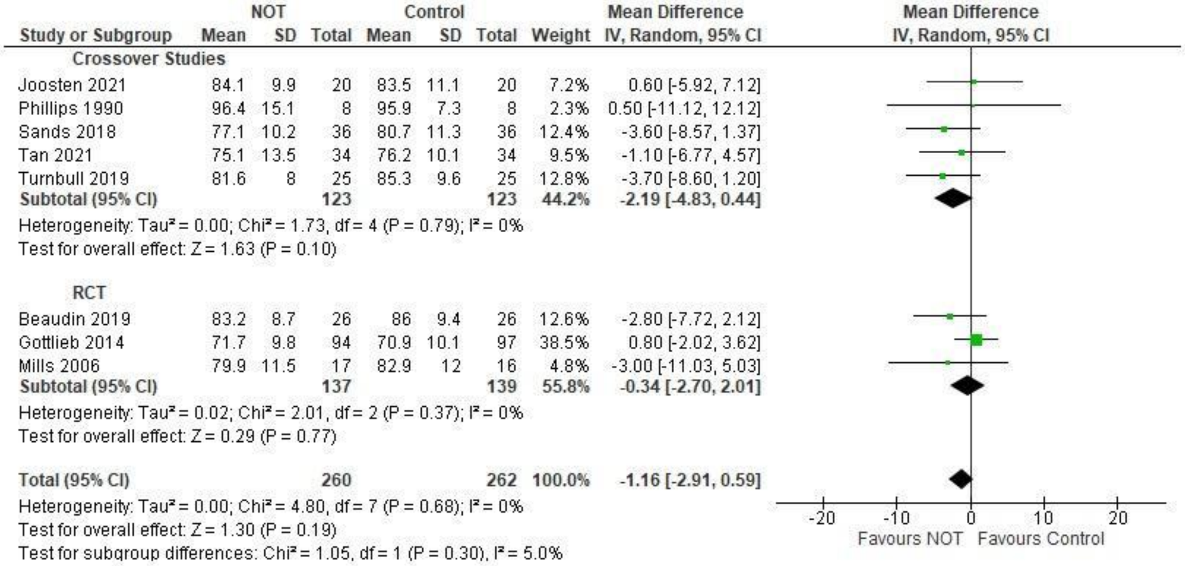

b) Based on oxygen flow rate

Study or Subgroup	NOT Mean	NOT SD	NOT Total	Control Mean	Control SD	Control Total	Weight	Mean Difference IV, Random, 95% CI
**2-3 L/min**								
Gottlieb 2014	71.7	9.8	94	70.9	10.1	97	38.5%	0.80 [-2.02, 3.62]
Mills 2006	79.9	11.5	17	82.9	12	16	4.8%	-3.00 [-11.03, 5.03]
Tan 2021	75.1	13.5	34	76.2	10.1	34	9.5%	-1.10 [-6.77, 4.57]
**Subtotal (95% CI)**			**145**			**147**	**52.7%**	**0.11 [-2.30, 2.52]**
Heterogeneity: Tau² = 0.00; Chi² = 0.98, df = 2 (P = 0.61); I² = 0%								
Test for overall effect: Z = 0.09 (P = 0.93)								
**More than 3L/min**								
Beaudin 2019	83.2	8.7	26	86	9.4	26	12.6%	-2.80 [-7.72, 2.12]
Joosten 2021	84.1	9.9	20	83.5	11.1	20	7.2%	0.60 [-5.92, 7.12]
Phillips 1990	96.4	15.1	8	95.9	7.3	8	2.3%	0.50 [-11.12, 12.12]
Sands 2018	77.1	10.2	36	80.7	11.3	36	12.4%	-3.60 [-8.57, 1.37]
Turnbull 2019	81.6	8	25	85.3	9.6	25	12.8%	-3.70 [-8.60, 1.20]
**Subtotal (95% CI)**			**115**			**115**	**47.3%**	**-2.58 [-5.12, -0.03]**
Heterogeneity: Tau² = 0.00; Chi² = 1.55, df = 4 (P = 0.82); I² = 0%								
Test for overall effect: Z = 1.98 (P = 0.05)								
**Total (95% CI)**			**260**			**262**	**100.0%**	**-1.16 [-2.91, 0.59]**

Heterogeneity: Tau² = 0.00; Chi² = 4.80, df = 7 (P = 0.68); I² = 0%
Test for overall effect: Z = 1.30 (P = 0.19)
Test for subgroup differences: Chi² = 2.26, df = 1 (P = 0.13), I² = 55.8%

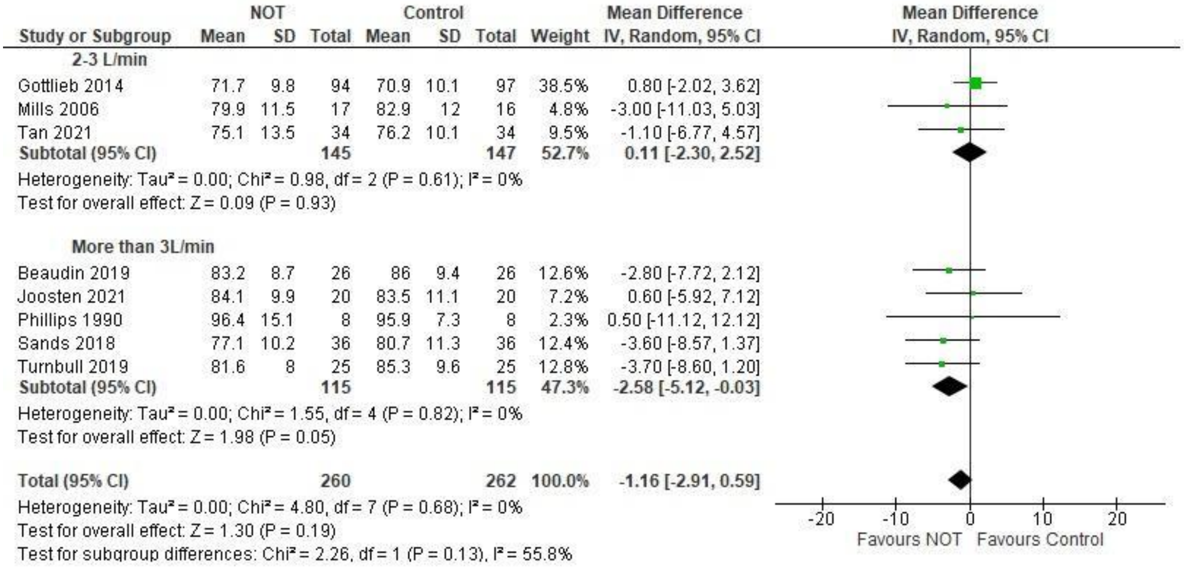

c) Based on duration of intervention

Study or Subgroup	NOT Mean	NOT SD	NOT Total	Control Mean	Control SD	Control Total	Weight	Mean Difference IV, Random, 95% CI
**1 night**								
Joosten 2021	84.1	9.9	20	83.5	11.1	20	7.2%	0.60 [-5.92, 7.12]
Sands 2018	77.1	10.2	36	80.7	11.3	36	12.4%	-3.60 [-8.57, 1.37]
Tan 2021	75.1	13.5	34	76.2	10.1	34	9.5%	-1.10 [-6.77, 4.57]
**Subtotal (95% CI)**			**90**			**90**	**29.1%**	**-1.74 [-4.98, 1.50]**
Heterogeneity: Tau² = 0.00; Chi² = 1.08, df = 2 (P = 0.58); I² = 0%								
Test for overall effect: Z = 1.05 (P = 0.29)								
**More than 1 night**								
Beaudin 2019	83.2	8.7	26	86	9.4	26	12.6%	-2.80 [-7.72, 2.12]
Gottlieb 2014	71.7	9.8	94	70.9	10.1	97	38.5%	0.80 [-2.02, 3.62]
Mills 2006	79.9	11.5	17	82.9	12	16	4.8%	-3.00 [-11.03, 5.03]
Phillips 1990	96.4	15.1	8	95.9	7.3	8	2.3%	0.50 [-11.12, 12.12]
Turnbull 2019	81.6	8	25	85.3	9.6	25	12.8%	-3.70 [-8.60, 1.20]
**Subtotal (95% CI)**			**170**			**172**	**70.9%**	**-0.92 [-3.00, 1.16]**
Heterogeneity: Tau² = 0.00; Chi² = 3.54, df = 4 (P = 0.47); I² = 0%								
Test for overall effect: Z = 0.86 (P = 0.39)								
**Total (95% CI)**			**260**			**262**	**100.0%**	**-1.16 [-2.91, 0.59]**

Heterogeneity: Tau² = 0.00; Chi² = 4.80, df = 7 (P = 0.68); I² = 0%
Test for overall effect: Z = 1.30 (P = 0.19)
Test for subgroup differences: Chi² = 0.18, df = 1 (P = 0.67), I² = 0%

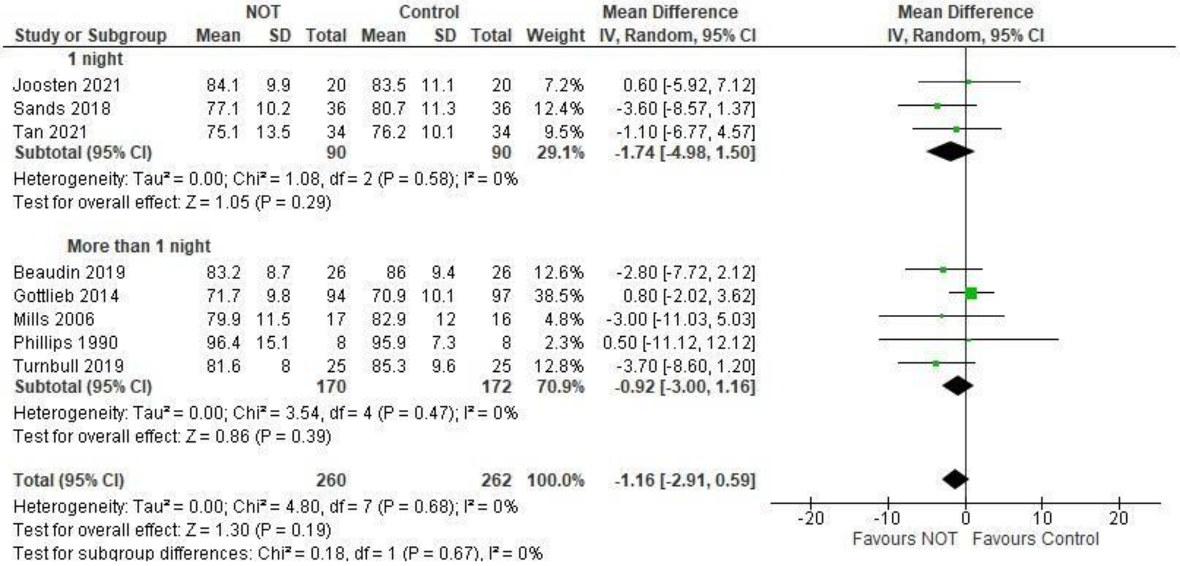

Supplementary Figure 5: Subgroup Analysis for Change in SBP

a) Based on study type

Study or Subgroup	NOT Mean	NOT SD	NOT Total	Control Mean	Control SD	Control Total	Weight	Mean Difference IV, Random, 95% CI
**Crossover Studies**								
Phillips 1990	-9.7	13.5	8	-4.7	14.5	8	3.6%	-5.00 [-18.73, 8.73]
Sands 2018	-0.8	8.4	36	3	11.2	36	26.7%	-3.80 [-8.37, 0.77]
Turnbull 2019	0.3	9.1	25	6.9	7.8	25	25.6%	-6.60 [-11.30, -1.90]
**Subtotal (95% CI)**			**69**			**69**	**55.9%**	**-5.15 [-8.34, -1.97]**
Heterogeneity: Tau² = 0.00; Chi² = 0.70, df = 2 (P = 0.70); I² = 0%								
Test for overall effect: Z = 3.17 (P = 0.002)								
**RCT**								
Beaudin 2019	2.1	13	26	1.5	12.6	26	13.1%	0.60 [-6.36, 7.56]
Gottlieb 2014	1.6	16.7	94	1.1	15.4	97	26.8%	0.50 [-4.06, 5.06]
Mills 2006	-5	14.4	17	-2.1	22.2	16	4.1%	-2.90 [-15.75, 9.95]
**Subtotal (95% CI)**			**137**			**139**	**44.1%**	**0.25 [-3.40, 3.91]**
Heterogeneity: Tau² = 0.00; Chi² = 0.25, df = 2 (P = 0.88); I² = 0%								
Test for overall effect: Z = 0.14 (P = 0.89)								
**Total (95% CI)**			**206**			**208**	**100.0%**	**-2.79 [-5.45, -0.14]**
Heterogeneity: Tau² = 1.43; Chi² = 5.72, df = 5 (P = 0.33); I² = 13%								
Test for overall effect: Z = 2.06 (P = 0.04)								
Test for subgroup differences: Chi² = 4.77, df = 1 (P = 0.03), I² = 79.0%								

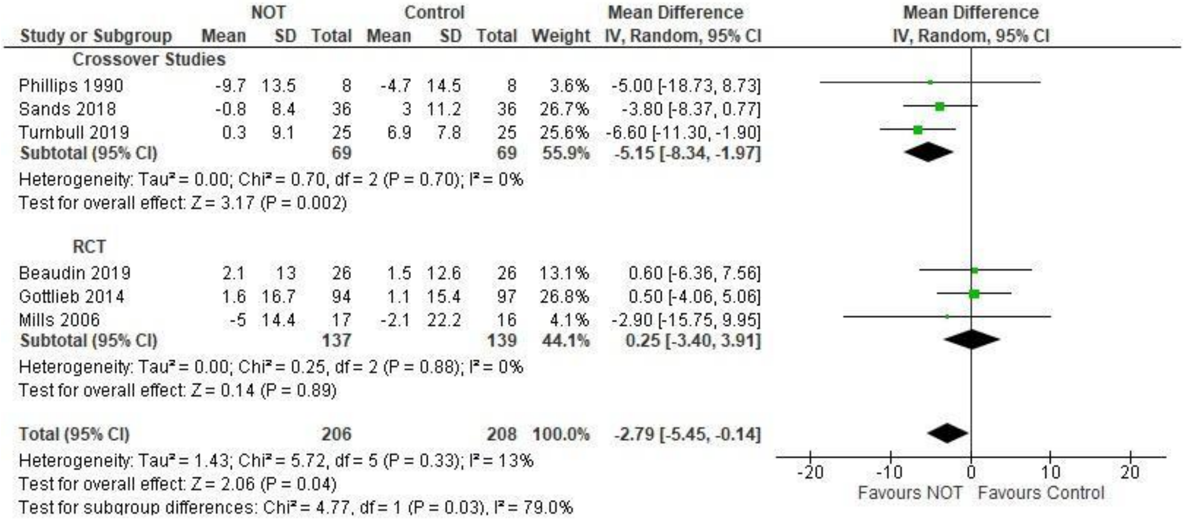

b) Based on oxygen flow rate

Study or Subgroup	NOT Mean	NOT SD	NOT Total	Control Mean	Control SD	Control Total	Weight	Mean Difference IV, Random, 95% CI
**2-3 L/min**								
Gottlieb 2014	1.6	16.7	94	1.1	15.4	97	26.8%	0.50 [-4.06, 5.06]
Mills 2006	-5	14.4	17	-2.1	22.2	16	4.1%	-2.90 [-15.75, 9.95]
**Subtotal (95% CI)**			**111**			**113**	**31.0%**	**0.12 [-4.18, 4.42]**
Heterogeneity: Tau² = 0.00; Chi² = 0.24, df = 1 (P = 0.63); I² = 0%								
Test for overall effect: Z = 0.05 (P = 0.96)								
**More than 3 L/min**								
Beaudin 2019	2.1	13	26	1.5	12.6	26	13.1%	0.60 [-6.36, 7.56]
Phillips 1990	-9.7	13.5	8	-4.7	14.5	8	3.6%	-5.00 [-18.73, 8.73]
Sands 2018	-0.8	8.4	36	3	11.2	36	26.7%	-3.80 [-8.37, 0.77]
Turnbull 2019	0.3	9.1	25	6.9	7.8	25	25.6%	-6.60 [-11.30, -1.90]
**Subtotal (95% CI)**			**95**			**95**	**69.0%**	**-4.16 [-7.05, -1.26]**
Heterogeneity: Tau² = 0.00; Chi² = 2.87, df = 3 (P = 0.41); I² = 0%								
Test for overall effect: Z = 2.81 (P = 0.005)								
**Total (95% CI)**			**206**			**208**	**100.0%**	**-2.79 [-5.45, -0.14]**
Heterogeneity: Tau² = 1.43; Chi² = 5.72, df = 5 (P = 0.33); I² = 13%								
Test for overall effect: Z = 2.06 (P = 0.04)								
Test for subgroup differences: Chi² = 2.61, df = 1 (P = 0.11), I² = 61.7%								

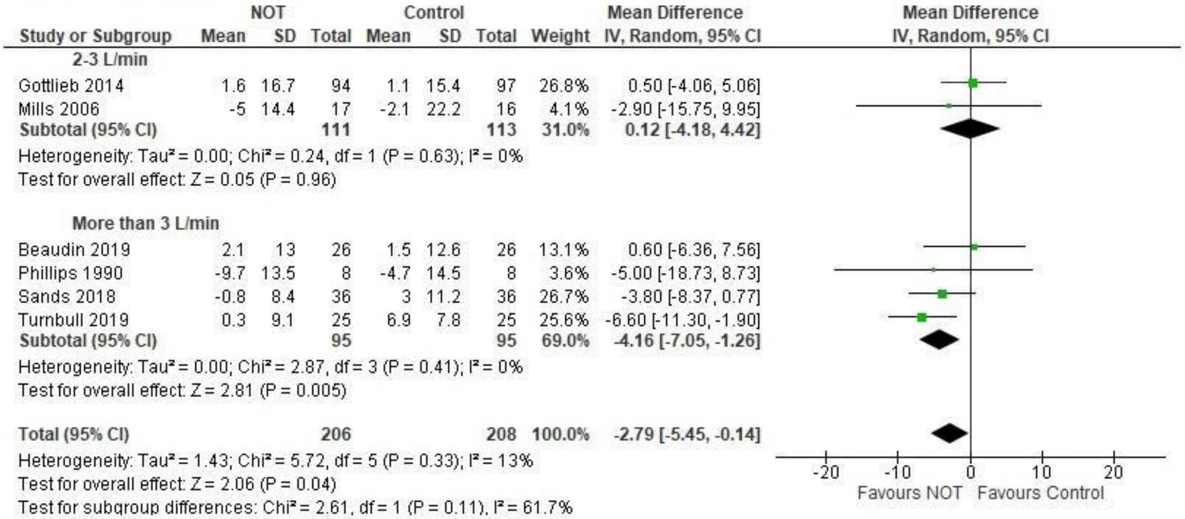

c) Based on duration of intervention

Study or Subgroup	NOT Mean	NOT SD	NOT Total	Control Mean	Control SD	Control Total	Weight	Mean Difference IV, Random, 95% CI
**More than 1 night**								
Beaudin 2019	2.1	13	26	1.5	12.6	26	19.7%	0.60 [-6.36, 7.56]
Gottlieb 2014	1.6	16.7	94	1.1	15.4	97	34.0%	0.50 [-4.06, 5.06]
Mills 2006	-5	14.4	17	-2.1	22.2	16	7.1%	-2.90 [-15.75, 9.95]
Phillips 1990	-9.7	13.5	8	-4.7	14.5	8	6.3%	-5.00 [-18.73, 8.73]
Turnbull 2019	0.3	9.1	25	6.9	7.8	25	32.9%	-6.60 [-11.30, -1.90]
**Subtotal (95% CI)**			**170**			**172**	**100.0%**	**-2.40 [-5.99, 1.19]**
Heterogeneity: Tau² = 4.45; Chi² = 5.48, df = 4 (P = 0.24); I² = 27%								
Test for overall effect: Z = 1.31 (P = 0.19)								
**Total (95% CI)**			**170**			**172**	**100.0%**	**-2.40 [-5.99, 1.19]**
Heterogeneity: Tau² = 4.45; Chi² = 5.48, df = 4 (P = 0.24); I² = 27%								
Test for overall effect: Z = 1.31 (P = 0.19)								
Test for subgroup differences: Not applicable								

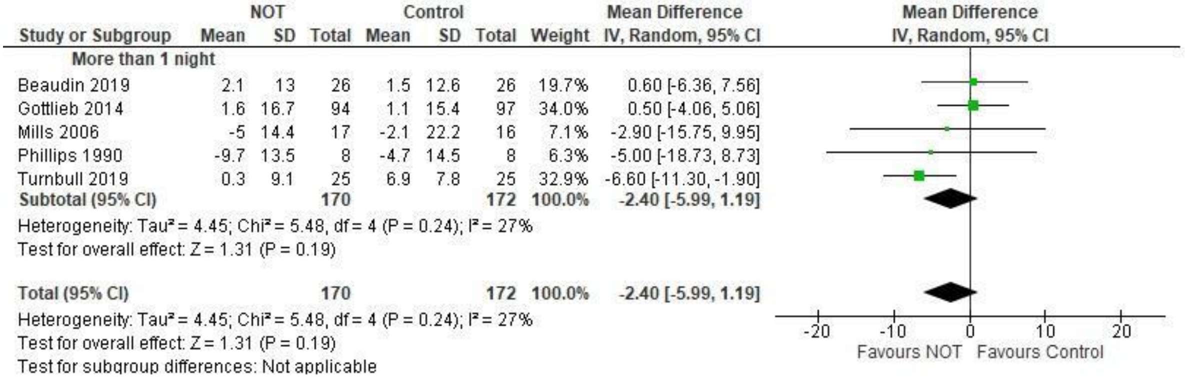

Supplementary Figure S6: Subgroup Analysis for Change in DBP

a) Based on study type

Study or Subgroup	NOT Mean	NOT SD	NOT Total	Control Mean	Control SD	Control Total	Weight	Mean Difference IV, Random, 95% CI
**Crossover Studies**								
Phillips 1990	-0.6	12.3	8	-1.1	7.9	8	2.6%	0.50 [-9.63, 10.63]
Sands 2018	0.9	5.4	36	4.1	9.2	36	21.9%	-3.20 [-6.68, 0.28]
Turnbull 2019	2.3	6.3	25	7	6.1	25	22.5%	-4.70 [-8.14, -1.26]
**Subtotal (95% CI)**			**69**			**69**	**47.0%**	**-3.71 [-6.09, -1.34]**
Heterogeneity: Tau² = 0.00; Chi² = 1.06, df = 2 (P = 0.59); I² = 0%								
Test for overall effect: Z = 3.06 (P = 0.002)								
**RCT**								
Beaudin 2019	0.7	9.1	26	3.4	9.8	26	10.1%	-2.70 [-7.84, 2.44]
Gottlieb 2014	0.9	9.1	94	1.3	9.4	97	38.6%	-0.40 [-3.02, 2.22]
Mills 2006	-1.4	9.7	17	-0.7	12.8	16	4.4%	-0.70 [-8.48, 7.08]
**Subtotal (95% CI)**			**137**			**139**	**53.0%**	**-0.86 [-3.10, 1.38]**
Heterogeneity: Tau² = 0.00; Chi² = 0.61, df = 2 (P = 0.74); I² = 0%								
Test for overall effect: Z = 0.75 (P = 0.45)								
**Total (95% CI)**			**206**			**208**	**100.0%**	**-2.20 [-3.83, -0.57]**
Heterogeneity: Tau² = 0.00; Chi² = 4.61, df = 5 (P = 0.47); I² = 0%								
Test for overall effect: Z = 2.65 (P = 0.008)								
Test for subgroup differences: Chi² = 2.93, df = 1 (P = 0.09), I² = 65.9%								

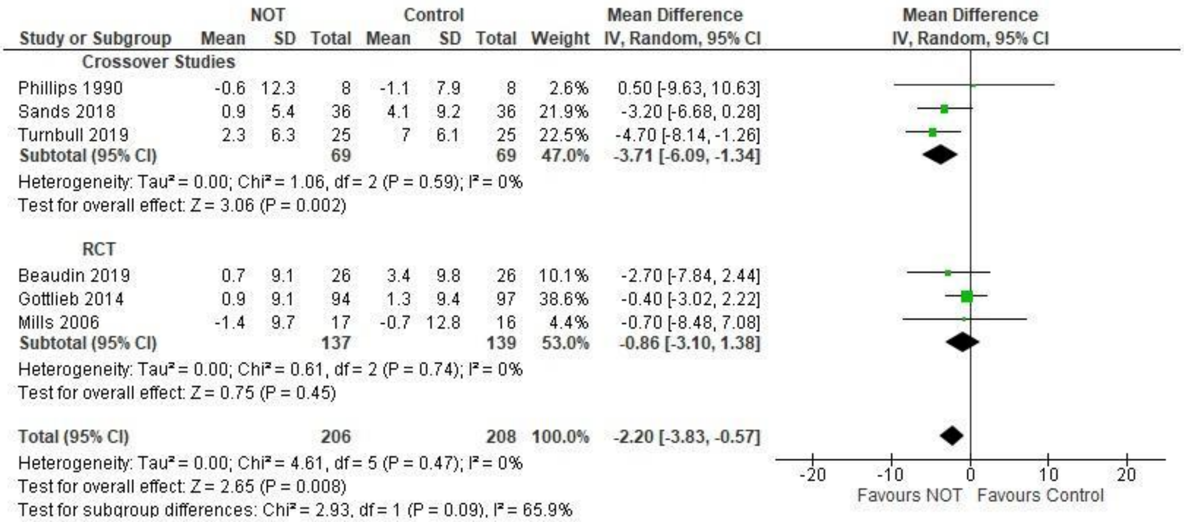

b) Based on oxygen flow rate

Study or Subgroup	NOT Mean	NOT SD	NOT Total	Control Mean	Control SD	Control Total	Weight	Mean Difference IV, Random, 95% CI
**2-3 L/min**								
Gottlieb 2014	0.9	9.1	94	1.3	9.4	97	38.6%	-0.40 [-3.02, 2.22]
Mills 2006	-1.4	9.7	17	-0.7	12.8	16	4.4%	-0.70 [-8.48, 7.08]
**Subtotal (95% CI)**			**111**			**113**	**43.0%**	**-0.43 [-2.92, 2.06]**
Heterogeneity: Tau² = 0.00; Chi² = 0.01, df = 1 (P = 0.94); I² = 0%								
Test for overall effect: Z = 0.34 (P = 0.73)								
**More than 3 L/min**								
Beaudin 2019	0.7	9.1	26	3.4	9.8	26	10.1%	-2.70 [-7.84, 2.44]
Phillips 1990	-0.6	12.3	8	-1.1	7.9	8	2.6%	0.50 [-9.63, 10.63]
Sands 2018	0.9	5.4	36	4.1	9.2	36	21.9%	-3.20 [-6.68, 0.28]
Turnbull 2019	2.3	6.3	25	7	6.1	25	22.5%	-4.70 [-8.14, -1.26]
**Subtotal (95% CI)**			**95**			**95**	**57.0%**	**-3.54 [-5.69, -1.38]**
Heterogeneity: Tau² = 0.00; Chi² = 1.19, df = 3 (P = 0.76); I² = 0%								
Test for overall effect: Z = 3.21 (P = 0.001)								
**Total (95% CI)**			**206**			**208**	**100.0%**	**-2.20 [-3.83, -0.57]**
Heterogeneity: Tau² = 0.00; Chi² = 4.61, df = 5 (P = 0.47); I² = 0%								
Test for overall effect: Z = 2.65 (P = 0.008)								
Test for subgroup differences: Chi² = 3.42, df = 1 (P = 0.06), I² = 70.7%								

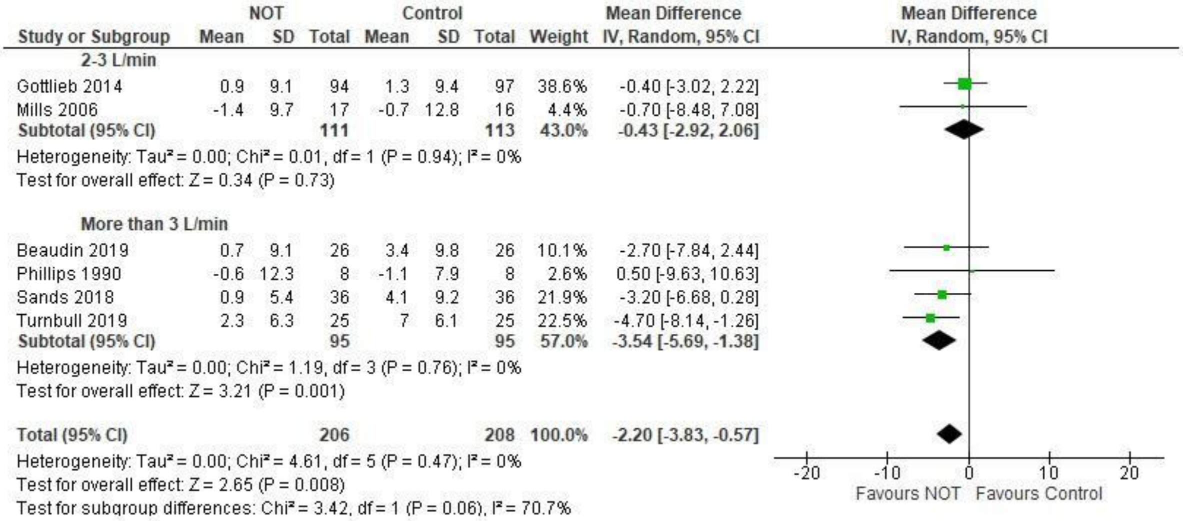

c) Based on duration of intervention

Study or Subgroup	NOT Mean	NOT SD	NOT Total	Control Mean	Control SD	Control Total	Weight	Mean Difference IV, Random, 95% CI
**More than 1 night**								
Beaudin 2019	0.7	9.1	26	3.4	9.8	26	13.7%	-2.70 [-7.84, 2.44]
Gottlieb 2014	0.9	9.1	94	1.3	9.4	97	47.4%	-0.40 [-3.02, 2.22]
Mills 2006	-1.4	9.7	17	-0.7	12.8	16	6.1%	-0.70 [-8.48, 7.08]
Phillips 1990	-0.6	12.3	8	-1.1	7.9	8	3.6%	0.50 [-9.63, 10.63]
Turnbull 2019	2.3	6.3	25	7	6.1	25	29.2%	-4.70 [-8.14, -1.26]
**Subtotal (95% CI)**			**170**			**172**	**100.0%**	**-1.96 [-3.90, -0.02]**
Heterogeneity: Tau² = 0.28; Chi² = 4.20, df = 4 (P = 0.38); I² = 5%								
Test for overall effect: Z = 1.98 (P = 0.05)								
**Total (95% CI)**			**170**			**172**	**100.0%**	**-1.96 [-3.90, -0.02]**
Heterogeneity: Tau² = 0.28; Chi² = 4.20, df = 4 (P = 0.38); I² = 5%								
Test for overall effect: Z = 1.98 (P = 0.05)								
Test for subgroup differences: Not applicable								

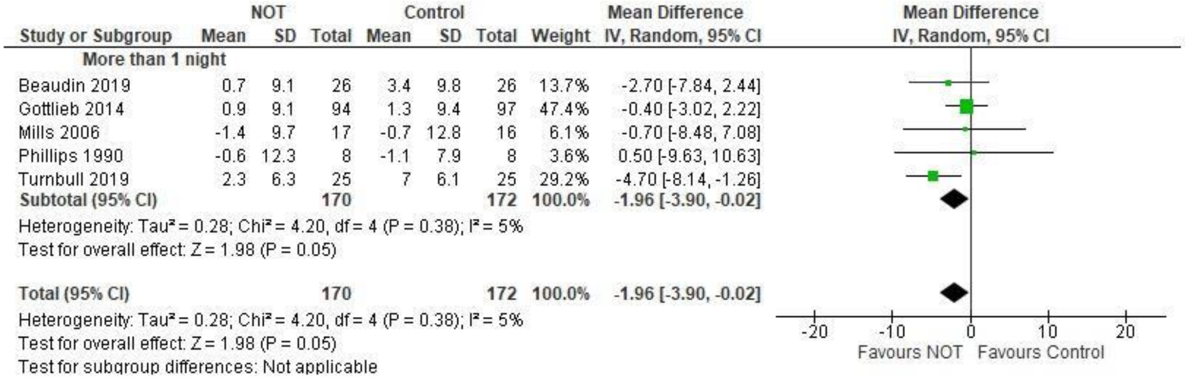

Supplementary Figure S7: Follow-up HR

Study or Subgroup	NOT Mean	NOT SD	NOT Total	Control Mean	Control SD	Control Total	Weight	Mean Difference IV, Random, 95% CI
Beaudin 2019	73.7	11.3	26	72.8	11.5	26	13.9%	0.90 [-5.30, 7.10]
Mills 2006	70.9	12.3	17	66.5	8.8	16	10.7%	4.40 [-2.86, 11.66]
Sands 2018	60.3	7.4	36	60.6	8.7	36	28.3%	-0.30 [-4.03, 3.43]
Tan 2021	65.8	8.9	34	70.2	7	34	27.6%	-4.40 [-8.21, -0.59]
Turnbull 2019	64.1	9.1	25	64.9	8.9	25	19.4%	-0.80 [-5.79, 4.19]
**Total (95% CI)**			**138**			**137**	**100.0%**	**-0.86 [-3.45, 1.74]**

Heterogeneity: $Tau^2 = 2.57$; $Chi^2 = 5.68$, df = 4 (P = 0.22); $I^2 = 30\%$
Test for overall effect: Z = 0.65 (P = 0.52)

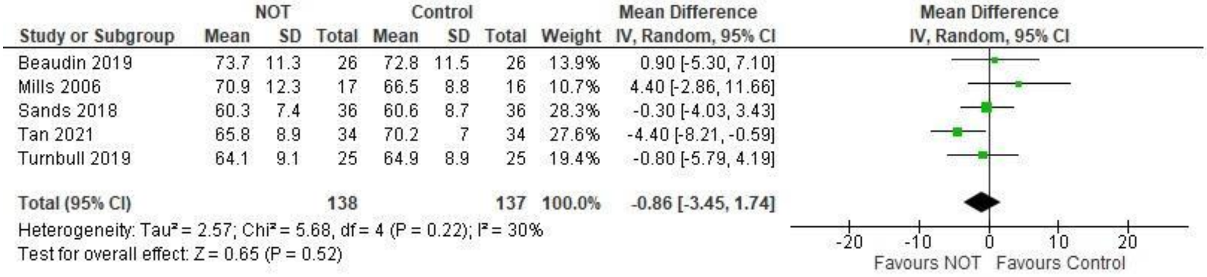

Supplementary Figure S8: Change in HR

Study or Subgroup	NOT Mean	NOT SD	NOT Total	Control Mean	Control SD	Control Total	Weight	Mean Difference IV, Random, 95% CI
Beaudin 2019	1	11.5	26	0.8	11.1	26	14.2%	0.20 [-5.94, 6.34]
Mills 2006	-1	11.9	17	0.1	7.7	16	11.6%	-1.10 [-7.90, 5.70]
Sands 2018	-2.2	8.5	36	-2.1	9	36	32.9%	-0.10 [-4.14, 3.94]
Turnbull 2019	3.2	5.2	25	2.3	7.6	25	41.3%	0.90 [-2.71, 4.51]
**Total (95% CI)**			**104**			**103**	**100.0%**	**0.24 [-2.08, 2.56]**

Heterogeneity: $Tau^2 = 0.00$; $Chi^2 = 0.30$, df = 3 (P = 0.96); $I^2 = 0\%$
Test for overall effect: Z = 0.20 (P = 0.84)

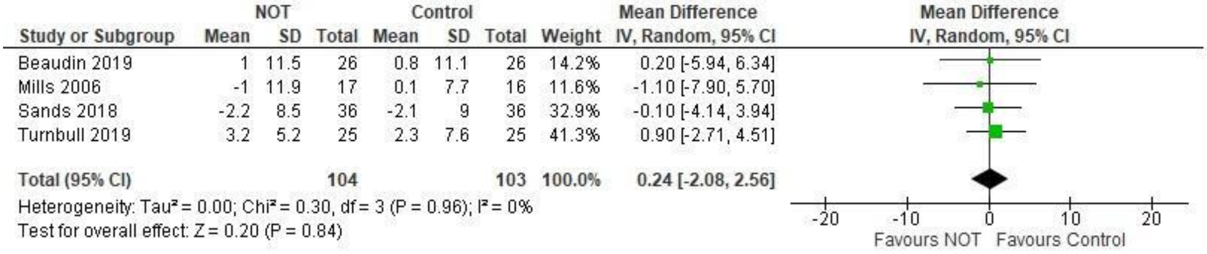

Supplementary Figure S9: Funnel plot for AHI with desataauration

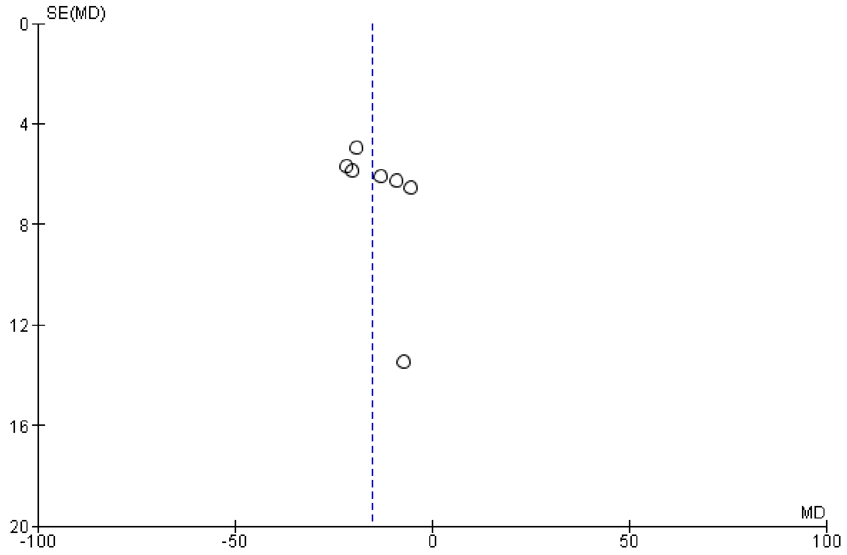

Supplementary Figure S10: Funnel plot for AHI without desaturation

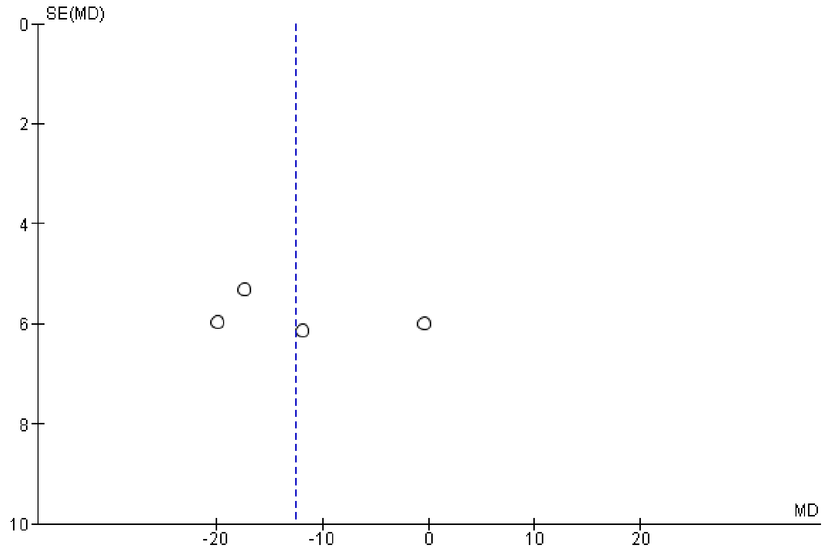

Supplementary Figure S11: Funnel plot for Oxygen Desaturation Index

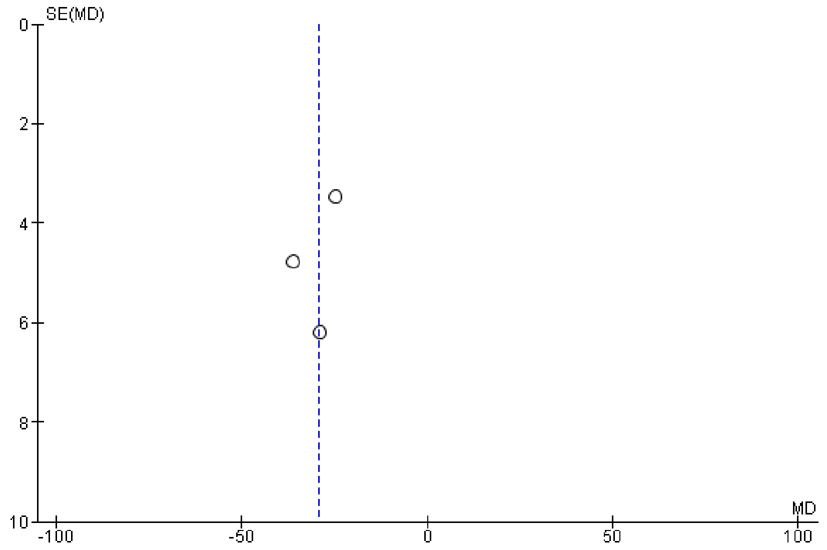

Supplementary Figure S12: Funnel plot for Arousal Index

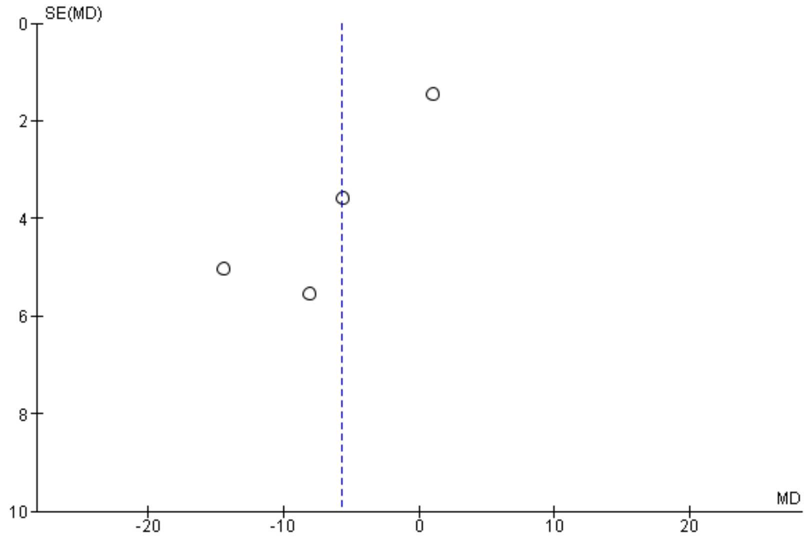

Supplementary Figure S13: Funnel plot for follow-up SBP

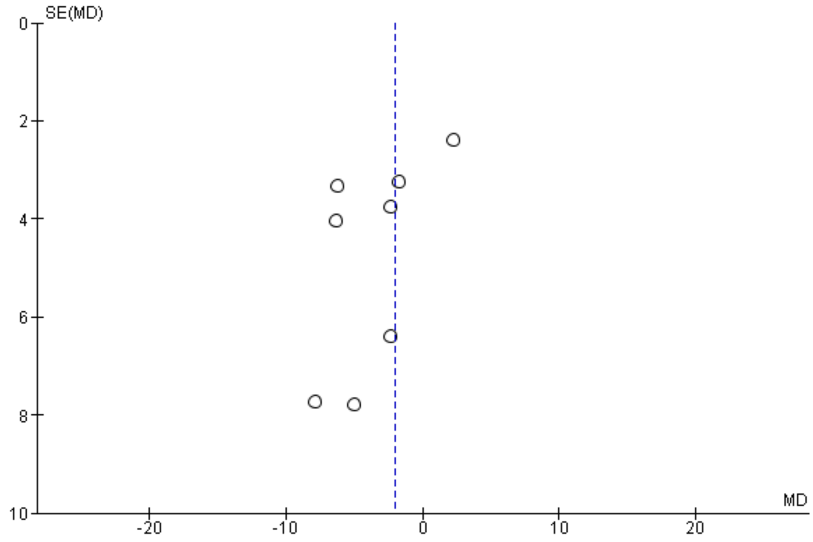

Supplementary Figure S14: Funnel plot for follow-up DBP

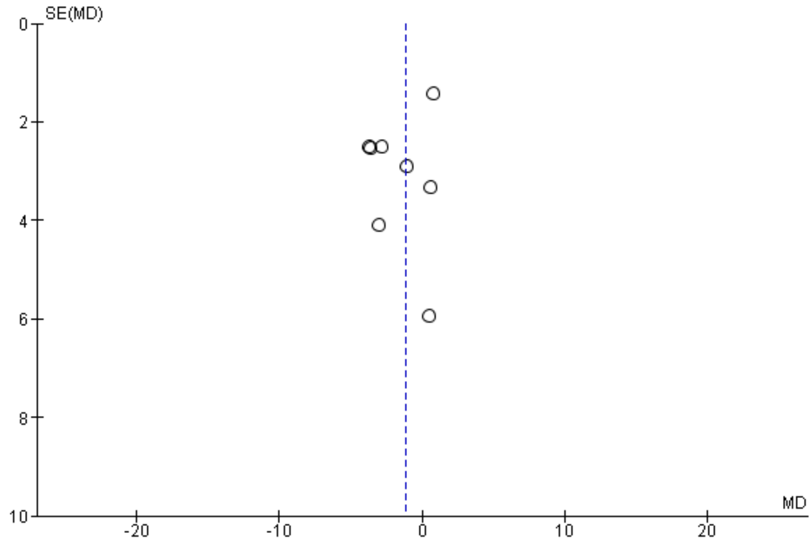

Supplementary Figure S15: Funnel plot for change in SBP

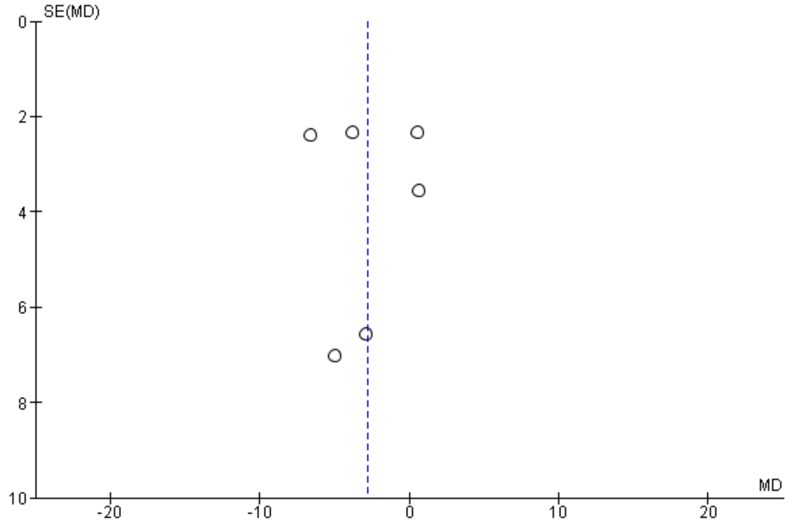

Supplementary Figure S16: Funnel plot for change in DBP

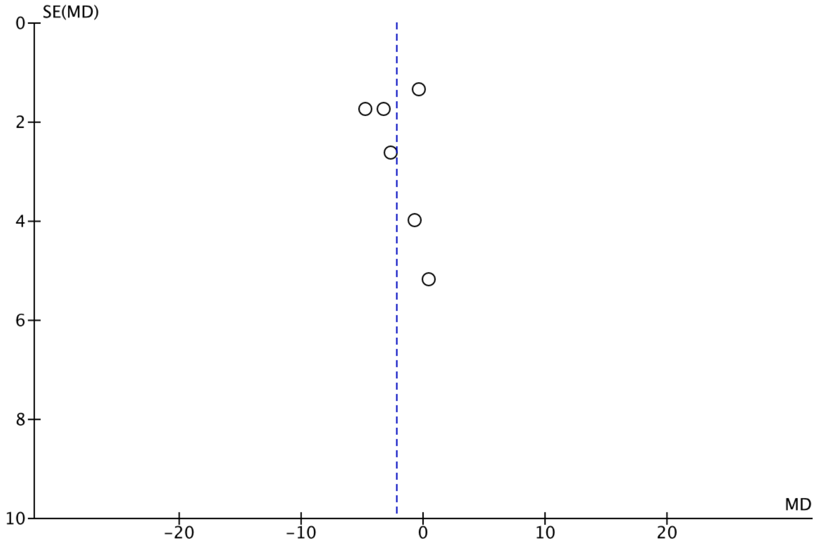

Supplement: Supplementary file 1 [file ERR-0173-2023.SUPPLEMENT.pdf]
